# A metagenomic approach to demystify the anaerobic digestion black box and achieve higher biogas yield: a review

**DOI:** 10.3389/fmicb.2024.1437098

**Published:** 2024-10-11

**Authors:** Iván Ostos, Luz Marina Flórez-Pardo, Carolina Camargo

**Affiliations:** ^1^Grupo de Investigación en Ingeniería Electrónica, Industrial, Ambiental, Metrología GIEIAM, Universidad Santiago de Cali, Cali, Colombia; ^2^Grupo de Investigación en Modelado, Análisis y Simulación de Procesos Ambientales e Industriales PAI+, Universidad Autónoma de Occidente, Cali, Colombia; ^3^Centro de Investigación de la Caña de Azúcar, CENICAÑA, Cali, Colombia

**Keywords:** methane, metagenome, microbiota, syntrophy, sequencing, DIET, GCM, SRM

## Abstract

The increasing reliance on fossil fuels and the growing accumulation of organic waste necessitates the exploration of sustainable energy alternatives. Anaerobic digestion (AD) presents one such solution by utilizing secondary biomass to produce biogas while reducing greenhouse gas emissions. Given the crucial role of microbial activity in anaerobic digestion, a deeper understanding of the microbial community is essential for optimizing biogas production. While metagenomics has emerged as a valuable tool for unravelling microbial composition and providing insights into the functional potential in biodigestion, it falls short of interpreting the functional and metabolic interactions, limiting a comprehensive understanding of individual roles in the community. This emphasizes the significance of expanding the scope of metagenomics through innovative tools that highlight the often-overlooked, yet crucial, role of microbiota in biomass digestion. These tools can more accurately elucidate microbial ecological fitness, shared metabolic pathways, and interspecies interactions. By addressing current limitations and integrating metagenomics with other omics approaches, more accurate predictive techniques can be developed, facilitating informed decision-making to optimize AD processes and enhance biogas yields, thereby contributing to a more sustainable future.

## Graphical abstract

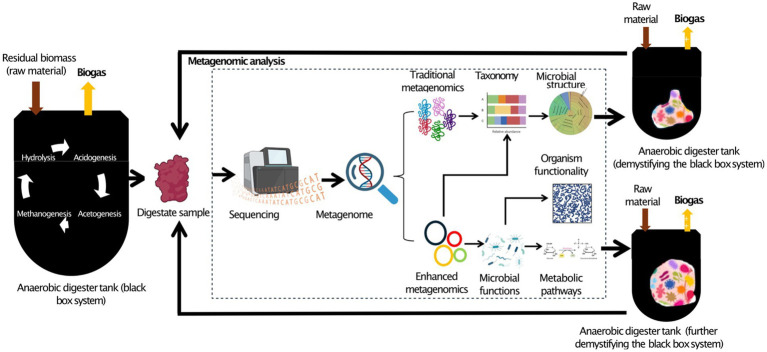

## 1 Introduction

Fossil fuels are the primary global energy source, substantially contributing to global warming while lacking replenishable capacity (Alengebawy et al., 2022). The rapid increase in residual biomass due to population growth, coupled with the urgent need to transition to renewable energy, has positioned anaerobic digestion (AD) for biogas production as a reliable technology for converting waste into energy (Subbarao et al., 2023; Piadeh et al., 2024).

Compared to solar and wind power, AD provides key advantages: (1) reliable baseload power, as it is not weather-dependent like solar and wind (Götz et al., 2016; Shah et al., 2024); (2) potentially lower investment and operation costs (Thi et al., 2016); (3) higher energy output per area once AD is stabilized (Araoye et al., 2018; Emrani and Berrada, 2024); and (4) flexibility in producing various products and byproducts (thermal and electrical power, vehicular fuel, biomethane, hydromethane, and green hydrogen; Villante and Genovese, 2012; Subbarao et al., 2023).

AD is a successful waste treatment method, reducing greenhouse gas emissions through microbial decomposition. The microbial community (MC) plays a key role in AD’s stability and biogas production, but limited knowledge of their roles and interactions hampers optimization efforts (Zhang et al., 2019b).

This review highlights metagenomics as a game-changer for AD, highlighting the importance of studying MC’s intricate composition, dynamics, and functions (Ghiotto et al., 2024b) to attain more efficient AD operations (Zhao et al., 2024).

This study emphasizes the advantages of metagenomics in understanding microorganisms’ taxonomy and function through innovative tools, showcasing microbiota’s often unnoticed yet crucial role during AD. Metagenomic enhancements allow for more accurate identification and comprehension of microbial ecological fitness, shared metabolic pathways, and interspecies interactions within AD, improving system knowledge (Schwan et al., 2020; Zhao et al., 2024). This review consolidates recent advances in AD research using metagenomics tools, highlighting key findings, addressing challenges, and outlining future research directions. It provides researchers insights to leverage metagenomics advancements, extending AD’s potential for biogas production and guiding future research toward more efficient AD approaches. Deepening the understanding of MC and their responses to reactor operational factors through metagenomics is expected to facilitate decision-making for higher CH_4_ content (Blades et al., 2017), contributing to the demystification of the AD black box.

## 2 Biogas composition and importance of the microbial community during AD

Before understanding microbial interactions, it is crucial to comprehend biogas composition and the roles of microorganisms in its production. As shown in Table 1, biogas primarily consists of methane (CH_4_) and carbon dioxide (CO_2_), with composition varying based on the substrate used. Substrate-specific levels of fats, carbohydrates, and proteins impact the biogas’ chemical nature and its impurity levels. These impurities include nitrogen (N₂), which dilutes CH_4_; ammonia (NH₃), inhibits methanogens and corrodes equipment; hydrogen sulfide (H₂S), a toxic compound that degrades biogas quality; oxygen (O₂), which can inhibit methanogens and create explosive mixtures with methane; hydrogen (H₂), an indicator of system imbalances; and carbon monoxide (CO), a toxic gas that can inhibit methanogenic activity at high concentrations.

**Table 1:** Typical biogas composition ranges from different substrates.

Substrate	Main products		Impurities						Reference
product	CH_4_%	CO_2_%	N_2_%	NH_3_%	H_2_S ppm	O_2_%	H_2_%	CO%	
Cow slurry	55–65	35–45	0–1	-	0–1%	0–2	0–1	0–3	Adebayo et al. (2015)
Landfill waste	50	15–50	0–5	1,394 ppm	5–60	1	0–5	115-500 ppm	Abedi et al. (2023)
	48–57	38-38	4.5–12	0.004–6.5	0.004–3.6%	1.8	-	0.001	Raboni and Viotti (2016), Sepúlveda et al. (2018)
Wastewater treatment plant	55–77	19–45	0–8	0-7 ppm	1–8×10^3^	0–2	-	0–0.01	Ong et al. (2014)
Agricultural waste	30–75	15–50	0–5	0-150 ppm	10–15,800	0–1	-	-	
	50–80	30–50	0–1	-	0–0.7%	0–1	0–2	0–1	Chen et al. (2015)
Non-defined	40-80	15–60	0–10	0–500	0–5,000	0–2	0–1	0–2	Ali Shah et al. (2014), Samuel (2015); Bharathiraja et al. (2018)

To fully comprehend the AD process and achieve biogas production, it is also essential to understand the roles of microorganisms and substrates in the four main phases: hydrolysis, acidogenesis, acetogenesis, and methanogenesis (Fedailaine et al., 2015; Xu et al., 2018b). Each phase is facilitated by specialized microbiota (Tijani et al., 2018; Xu et al., 2018b; Table 2).

**Table 2:** Typical microorganisms associated with the four phases in anaerobic digestion.

Phase in AD	Product transformed	Microorganisms involved	Product obtained	Reference
1. Hydrolysis	Cellulose	*Acetovibrio microbispora, Clostridium cellobioporus, Clostridium lochhadii, Clostridium stercorarium, Clostridium thermocellum, Micromonospora bispora, Ruminococcus albus, Ruminococcus Bacteroides, and Ruminococcus flavefaciens*	Amino acids, sugars, and organic fatty acids	Tabatabaei et al. (2020); Chukwuma et al. (2021)
	Hemicellulose	*Bacteroides fibrisolvens, Bacteroides ruminicola, Ruminococcus albus, Ruminococcus flavenfaciens, and Paenibacillus polymyxa*		Krishna and Kalamdhad (2014); Chukwuma et al. (2021)
	Lignocellulose	*Thermobifida fusca, Clostridium thermocellum, Caldicellulosiruptor bescii, Acinetobacter* sp.*, Klebsiella variicola, Bacillus* sp.*, Proteus mirabilis, Stenotrophomonas maltophilia, and Chryseobacterium gleum*		Chukwuma et al. (2021)
2. Acidogenesis	Amino acids, sugars, and organic fatty acids	*Bacillus, Escherichia coli, Lactobacillus, and Salmonella, Streptococcus*	Short-chain organic fatty acids and alcohols such as methanol, ethanol, and aldehydes. NH_3_ and H_2_S	Ali Shah et al. (2014), Tabatabaei et al. (2020)
3. Acetogenesis	Short-chain organic fatty acids and alcohols	*Clostridium, Acetobacterium, Syntrophomonas, Syntrophobacter, Sporomusa, Syntrophospora, Thermosyntropha, Ruminococcus, and Eubacterium*	H_2_, CO_2_ and acetate	Mehariya et al. (2018); Tabatabaei et al. (2020)
4. Methanogenesis	Acetate and H_2_	Archaea: *Methanobacterium, Methanothermobacter, Methanospirillum, Methanobrevibacter, Methanoculleus, Methanothrix (previously known as Methanosaeta), Methanosarcina, Methanolobus, and Methanococcus*	CH_4_ and CO_2_	Tabatabaei et al. (2020); Zhang X. et al. (2023)

The initial phase, hydrolysis, can limit the overall process rate (Krishna and Kalamdhad, 2014). During this stage, hydrolytic microbes (Table 2) enzymatically break down complex polymers such as lipids, proteins, and carbohydrates into smaller molecules such as amino acids, sugars, and fatty acids (Bharathiraja et al., 2018; Khanh Nguyen et al., 2021). Cellulose is degraded by organisms such as *Clostridium*, *Cellulomonas*, *Bacillus*, *Thermomonospora*, *Ruminococcus*, *Bacteroides*, *Erwinia*, *Acetovibrio*, and *Microbispora* (Chukwuma et al., 2021), while hemicellulose is targeted by *Bacteroides*, *Ruminococcus* (Krishna and Kalamdhad, 2014), and *Streptomyces* genus, associated with high ratios of metabolite production and biotransformation, even capable of degrading lignocellulose (de Lima Procópio et al., 2012). Lignin degradation is performed by *Streptomyces*, *Sphingomonas*, *Pseudomonas*, *Rhodococcus*, and *Nocardia* with *Clostridium thermocellum* and *Caldicellulosiruptor bescii*, also breaking lignin down while utilizing sugars(Chukwuma et al., 2021). Organisms such as *Acinetobacter* sp., *K. variicola, Bacillus* sp., *P. mirabilis*, and *S. maltophilia* are considered lignocellulose degraders (Chukwuma et al., 2021).

Later, hydrolysis products are ingested by acidogenic bacteria to produce short-chain volatile fatty acids (VFAs) and alcohols (Table 2; Ali Shah et al., 2014; Tabatabaei et al., 2020; Zhang X. et al., 2023). In acidogenesis, NH₃ and H₂S have a significant impact since they produce an obnoxious odor associated with system inhibition and gear corrosion (Ali Shah et al., 2014).

During acetogenesis, acids are converted into H_2_, CO_2,_ and acetic acid through acetogenic bacteria (Table 2; Mehariya et al., 2018; Tabatabaei et al., 2020). Acetic acid is an important product as it can be used by methanogenic organisms to obtain CH_4_. In this phase, *Methanobacterium* spp. can break down valeric acid to propionic acid, to be later turned into acetic acid by archaea such as *Methanobacterium propionicum* (Ali Shah et al., 2014). Moreover, released H_2_ from acetogenesis can be exploited through a symbiotic relationship (syntrophy) between acetogenic bacteria and autotrophic methane bacteria (Li et al., 2018; Tijani et al., 2018). This stage is accountable for producing approximately 70% of methane, 25% of acetates, and 11% of hydrogen, making acetates a crucial intermediate for AD mechanisms (Ali Shah et al., 2014; Jain et al., 2015).

During methanogenesis, the final stage of conventional AD, acetate, and H_2_ are converted into CH_4_ and CO_2_ by methanogenic, hydrogenotrophic, and acetoclastic archaea (Table 2; Manjusha and Beevi, 2016; Tabatabaei et al., 2020). Approximately 30% of CH_4_ comes from CO_2_ reduction by autotrophic methane bacteria, resulting in H_2_ depletion (Ali Shah et al., 2014). Low pH values from VFAs and significant production of H_2_S could inhibit methanogenesis (Izumi et al., 2010).

Optimizing biogas production in AD requires a deep understanding of MC structures due to their enormous role in the process. Research should focus on substrate composition, MC dynamics, metabolic networks, and their impact on performance. Metagenomic analysis offers a powerful method for quantifying and identifying ecological niches in complex communities (Zhang Y. et al., 2019; Zhu et al., 2019). Despite its importance, replication is often overlooked owing to cost and time constraints, impacting the robustness of findings in microbial ecology (Prosser, 2010).

## 3 Metagenomic analysis

Metagenomics is a revolutionary method for exploring microbial ecosystems and uncovering complex microbiological interactions (Taş et al., 2021). Shotgun sequencing typically allows for the analysis of total DNA from a microbiome (Lindner et al., 2024). In AD research, metagenomics focuses on studying genetic material from feedstocks or digestates to identify and examine MCs’ structure, abundance, functionality, and interactions using phylogenetic analysis and DNA sequencing (Bedoya et al., 2019; Zhang et al., 2019a). This information can be correlated with operational factors to reveal insights into biogas production and the essential role of microbiota in AD processes.

**Figure 1:**
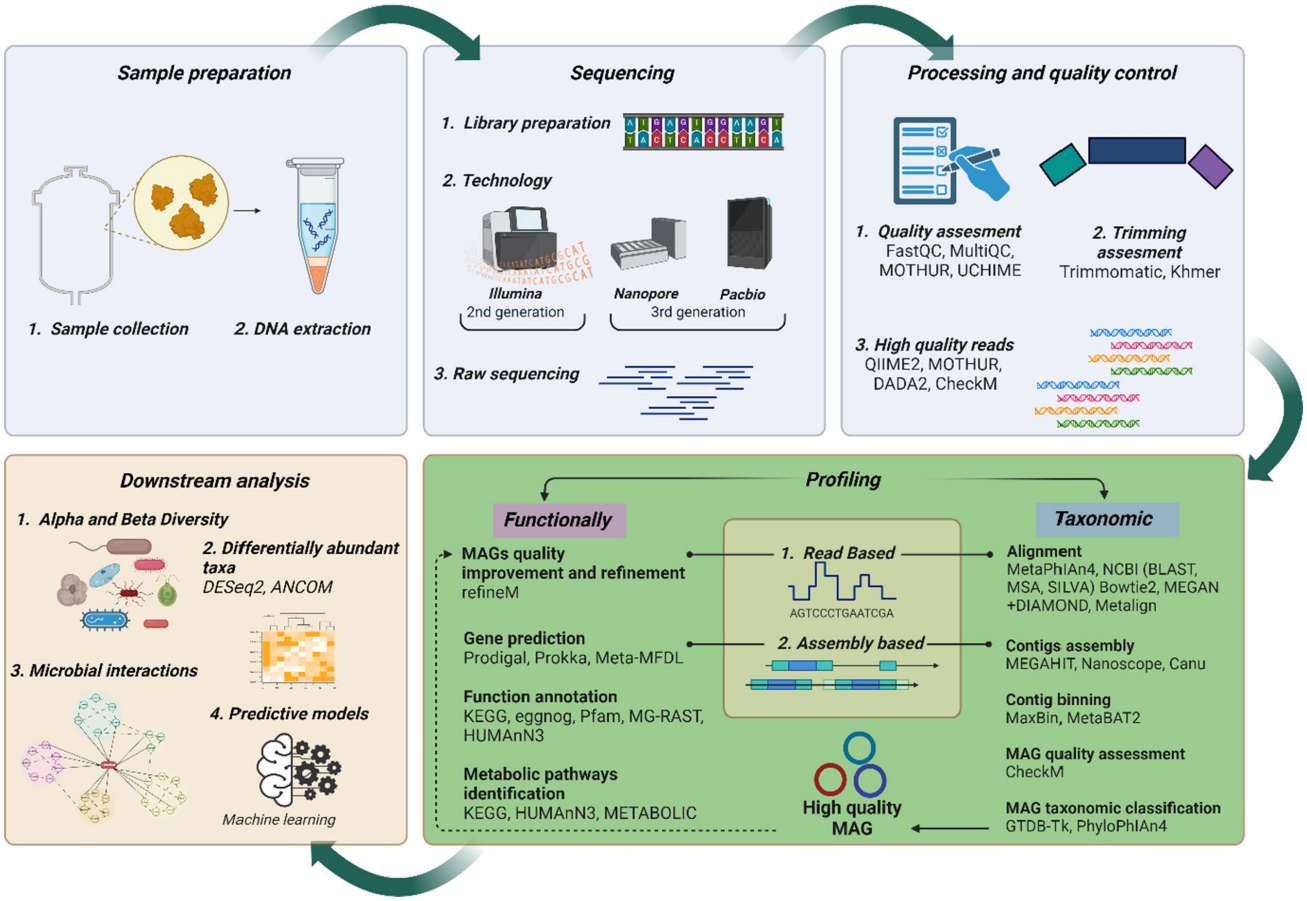
Current metagenomics data analysis flow chart. Created with BioRender.com.

Figure 1 illustrates a streamlined metagenomic data analysis workflow, from sample collection to downstream analysis. Sequencing options include short-read (second generation), long-read (3^rd^ generation), or hybrid approaches for improved accuracy and completeness (Chen et al., 2020; Eisenhofer et al., 2024). After sequencing, processing, and quality control, ensure data quality. Taxonomic and functional analysis follows, with amplicon sequencing typically identifying MCs at the genus level, while advanced metagenomics can achieve strain-level resolution in some cases. This broadens perceptions of microbial interactions and ecological dynamics. Advanced metagenomics also reveals novel functions and interactions, surpassing traditional approaches limited to DNA amplification. Downstream analysis includes diversity assessment, differential abundance, and network analysis, resulting in deeper insights into the AD microbiome. These findings can input ML predictive models to increase biogas yields.

### 3.1 Sample preparation

This early quality control step aims to minimize false positives. Meticulous sampling and cleaning are crucial to prevent interference during collection. Repeated sampling helps establish consistency and minimizes experimental error.

While no single formula guarantees successful DNA extraction, proper cleaning, lysing, extraction, purification, and quality assessment are needed before metagenomics analysis. Errors in these steps can result in unreliable sequencing. In AD, the MP Biomedicals FastDNA SPIN kit for soil is preferred for its high-quality DNA yield and effective lysis, ensuring accurate microbiome representation. Other commonly used kits include QIAGEN DNeasy PowerSoil Pro, Omega Bio-Tek E.Z.N.A. Soil DNA, and ZymoBIOMICS DNA Miniprep.

Library preparation is essential for DNA sequencing and analysis, permitting the identification and characterization of MCs. Illumina MiSeq and Nextera are widely used systems for this purpose.

### 3.2 Sequencing technologies

#### 3.2.1 Second generation

Next-generation sequencing (NGS), or second-generation sequencing, works with short DNA reads between 50 and 400 base pairs, simultaneously enabling high-throughput, parallel sequencing of multiple DNA strands.

##### 3.2.1.1 16S rRNA-based metagenomics or metataxonomics

The 16S rRNA gene is a fundamental tool in microbial analysis, traditionally grouped into operational taxonomic units but now clustered into amplicon sequence variants for greater accuracy. As a conserved phylogenetic marker across diverse microbial lineages, it allows for organism clustering, with conservation levels determining taxonomic resolution (Case et al., 2007; Yang et al., 2016). Its conserved nature and cost-effective sequencing make it popular in AD research (Zhang et al., 2019a). Nonetheless, the 16S rRNA gene cannot differentiate closely related species and does not provide functional or metabolic comprehension (Golub et al., 2011). Therefore, advanced metagenomics, transcriptomic (gene expression), proteomics (protein production), and metabolomic (metabolite profiles) are necessary to understand AD microbiome function and metabolism more deeply (Tabatabaei et al., 2020).

Shotgun metagenomic sequencing, which sequences the entire genomic DNA in a sample, provides a comprehensive view of the metagenome and insights into the microbiota’s functional potential (Portik et al., 2022). In contrast, amplifying and sequencing taxonomic marker genes, like the 16S rRNA gene, is better termed “metataxonomic” since it focuses on specific regions rather than the entire genome (Marchesi and Ravel, 2015; Portik et al., 2022; Lindner et al., 2024).

DNA polymerases, which replicate DNA by adding nucleotides to the 3′-end of an existing strand, require primers, short single-stranded nucleic acids, to anneal to complementary sequences on the DNA template, granting starting points (Michael et al., 2015; Abellan-Schneyder et al., 2021). Table 3 lists commercial primers for 16S rRNA gene amplification used in AD microbiota studies, highlighting their strengths and weaknesses. Most primers target the V3-V4 (Cattaneo et al., 2022; Alghashm et al., 2023; Zhao et al., 2023) and V4-V5 (Alghashm et al., 2023; Li et al., 2023; Chen et al., 2024) regions, which are informative for identifying bacteria and archaea.

**Table 3:** Commonly used primers in AD.

Forward primer	Reference	Reverse primer	Reference	Strengths	Weaknesses
338F	Li et al. (2022); Pei et al. (2022); Alghashm et al. (2023); Wei et al. (2023); Zhao et al. (2023)	806R	Li et al. (2022, 2023); Alghashm et al. (2023); Zhao et al. (2023); Liu et al. (2024)	High bacteria diversity coverage	Able to amplify non-targeted sequences
340F	Song et al. (2021); Sun Z. et al. (2023); Rao et al. (2024)	907R	Alarcón-Vivero et al. (2022); Chen et al. (2024); Cheng et al. (2024)	Good resolution for taxonomic identification	Lower archaea coverage
341F	Cattaneo et al. (2022); Khanthong et al. (2023); Sun Z. et al. (2023); Liu et al. (2024)	1000R	Song et al. (2021); Sun Z. et al. (2023); Rao et al. (2024)	High specificity for bacteria and archaea	Low abundance taxa might get missed
349F	Song et al. (2021); Sun Z. et al. (2023); Rao et al. (2024)	1059R	Mao et al. (2021); Rhee et al. (2021); Khanthong et al. (2023); Zhang et al. (2024)	Recommended for varied microbiota	Bias may be encountered in some microbiota
787F	Mao et al. (2021); Rhee et al. (2021); Khanthong et al. (2023); Zhang et al. (2024)	1492R	McAteer et al. (2020); Mao et al. (2021); Trego et al. (2021); Zhang et al. (2024)	Wide range bacteria coverage	Not great coverage for archaea
1369F	McAteer et al. (2020); Mao et al. (2021); Trego et al. (2021); Zhang et al. (2024)			Targets conserved regions	Resolution for closely related species is a low

The following are examples of frequently and recently used sets of primers regarding AD studies: 338F/806R (Li et al., 2022; Alghashm et al., 2023; Wei et al., 2023), 787F/1059R (Mao et al., 2021; Khanthong et al., 2023; Zhang et al., 2024), and 1369F/1492R (Mao et al., 2021; Trego et al., 2021; Zhang et al., 2024).

Primer selection is critical in AD research because potential primer bias can affect MC representation by preferentially amplifying certain sequences. The 338F/806R primer is widely used to capture the diverse microbial participants in AD. Other primers, like 787F/1059R and 1369F/1492R, are also employed but may have limitations, such as insufficient archaea coverage or taxonomic resolution, impacting MC analysis. Therefore, selecting primers should align with the specific research goals and AD systems through validation to ensure accuracy and reliability.

##### 3.2.1.2 Shotgun sequencing

Shotgun sequencing is a leading metagenomic technique for characterizing the genetic composition of complex microbiomes, providing taxonomic and functional insights by analyzing the entire DNA content of an MC. Unlike amplicon sequencing, it allows detailed genome reconstruction through metagenomics assembly and binning, which is beneficial for understanding microbial dynamics.

Traditional reference-based assembly is effective for known organisms but struggles with novel or uncultured microbes common in AD environments. *De novo* assembly addresses this by reconstructing genomes directly from sequencing data without a reference genome, a significant advance in the field of identifying unknown species.

During assembly, short DNA sequences (reads) are combined into longer contiguous sequences (contigs) and then into scaffolds, often using paired-end reads or long-read data to enhance genome completeness. Binning groups these scaffolds into metagenome-assembled genomes (MAGs), providing detailed insights into microbial functions in AD (Lischer and Shimizu, 2017; Hauptfeld et al., 2024). However, short-read sequences can hinder genome assembly due to limitations in resolving repetitive sequences. Long-read technology inclusion improves genome assembly quality and resolution (Xie et al., 2020; Becker et al., 2023).

Despite its benefits, shotgun sequencing faces challenges such as short read length, missing data, and sequencing errors. Combining reference mapping with *de novo* assembly could enhance genome reconstruction by leveraging information from related genomes, offering a more robust understanding of microbial processes in AD (Lischer and Shimizu, 2017).

#### 3.2.2 Third generation

Unlike short reads that usually cover a single gene portion, long reads can span multiple genes, making them more effective for sequence alignment and genome matching (Portik et al., 2022). Third-generation sequencing (TGS), such as those by Pacific Biosciences (PacBio) and Oxford Nanopore Technologies (ONT), represent significant advancements in sequencing, generating long reads without PCR amplification, thus reducing bias and improving genome coverage (Xiao and Zhou, 2020; Athanasopoulou et al., 2021). This gives TGS proven advantages over second-generation platforms in analyzing complex MCs.

For instance, in biogas research, TGS facilitates in-depth analysis of MCs within AD systems, which is essential for optimizing biogas production processes. TGS can provide a clear picture of microbial diversity and interactions, aiding in the identification of key microorganisms and their functional roles, which allows for targeted interventions to improve AD systems’ stability and efficiency (Xie et al., 2020).

Even though long TGS reads have accuracy issues, they may provide proper overall taxonomic classification because of the higher information content per read (Brandt et al., 2020). TGS requires careful sequencing library preparation to achieve optimal quantitative metagenomic analysis, enabling researchers to effectively correlate metagenomic data with biogas process operation.

PacBio’s single-molecule real-time technology produces highly accurate long reads, with the Sequel IIe system generating up to 4 million reads in a 30-h run, improving metagenomic assemblies and genome binning, thanks to its advanced data processing capacity, which reduces the computational cost and facilitates faster data transfer (Athanasopoulou et al., 2021). Comparative metagenomic analysis using short and long-read assemblers in AD systems indicates that PacBio long reads improve metagenomic assemblies, enhance gene catalog statistics, refine genome binning, and enhance the functional understanding of microbiomes (Xie et al., 2020).

ONT, which uses protein nanopores embedded in an electrically resistant membrane to sequence single-stranded DNA or RNA molecules (Cuber et al., 2023), has transformed sequencing by introducing pocket-sized devices capable of real-time sequencing in the field, facilitating immediate analysis of environmental samples and potential reactor monitoring (Athanasopoulou et al., 2021). The use of automated, biogas reactor-specific monitoring tools, such as those employing ML, could help predict operational problems (Brandt et al., 2020).

Tools such as LongQC assess TGS data quality, while Canu, Prowler, and Porechop are used for adapter trimming. Minimap2, GraphMap, and BWA-MEM are widely used aligners for TGS data, offering robust performance for aligning long reads (Wick et al., 2017a; Athanasopoulou et al., 2021). Additionally, Flye is effective for genome assembly, while tools such as Quiver, Nanopolish, and Racon are suitable for read corrections and polishing, enhancing the accuracy of TGS-based assemblies (Vaser et al., 2017; Kolmogorov et al., 2019).

The major drawback of these technologies is the high error rate, approximately 15%, observed during sequencing, making TGS less ideal for accurate detection (Athanasopoulou et al., 2021). However, improvements in sequencing chemistry are expected to reduce these errors and enhance accuracy. Despite this, the development of necessary bioinformatics tools and pipelines for TGS data analysis remains challenging, limiting the full exploitation of the vast amount of data produced.

#### 3.2.3 Hybrid sequencing

Hybrid sequencing approaches, which combine short-read and long-read technologies, offer a cost-effective strategy for generating comprehensive genome assemblies, balancing the strengths and weaknesses of each method (Eisenhofer et al., 2024). For instance, the Unicycler pipeline uses Illumina short reads for initial assembly and ONT long reads to close gaps and resolve repeats, often producing complete genome assemblies. These hybrid assemblies are highly accurate and complete, consistent with reference genomes in size and GC content, and capable of producing high-quality MAGs that reveal the functional potential of AD microbiomes, including unknown bacteria and archaea (Chen et al., 2020).

A study by Singleton et al. (2021) combined long and short reads to assemble over 1,000 high-quality MAGs from anaerobic conditions, identifying key functional microbes, such as the novel genus *Ca. methylophosphatis*. This highlights the importance of raising standards for MAG quality to facilitate the identification and experimental validation of functional microbes while avoiding contamination with low-quality MAGs.

In AD microbiomes, hybrid approaches are becoming increasingly important for analyzing system stability and buffer capacity (Becker et al., 2023). These methods have enabled the identification of potentially new organisms critical to AD processes, such as *Syntrophobacteraceae* (involved in short-chain fatty acid oxidation), *Syntrophomonadaceae* (butyrate oxidation), and *Methanoculleus* (hydrogenotrophic methanogenesis; Becker et al., 2023).

In summary, hybrid sequencing is revolutionizing the field of AD metagenomics by generating high-quality MAGs and deepening the understanding of MC and their functions. As sequencing technologies and computational power continue to advance, these hybrid approaches will further enhance the investigation of AD microbiomes, leading to improved outcomes and expanding microbial knowledge.

### 3.3 Processing and quality control

After sequencing, data are subjected to rigorous QC and trimming. Tools such as MultiQC, Mothur, FastQc, and UCHIME assess sequencing quality and identify low-quality reads and contamination. Trimming tools such as Trimmomatic remove low-quality bases and adapter sequences, while K-mer-based tools (substrings of constant length k that capture consecutive bases, enabling efficient sequence comparison and analysis) like UCHIME identify and remove chimeric sequences. Trimming must be carefully balanced, as overly aggressive trimming can lead to data loss and less accurate assemblies (Yang et al., 2019; Taş et al., 2021; Häntze and Horton, 2023). The combination of QC and trimming is crucial to avoid biases in taxonomic profiling and functional annotation, as downstream accuracy relies on the input data quality.

Since QIIME2 performs initial QC and trimming, it plays a crucial role in downstream analysis, offering taxonomic and functional profiling tools, diversity analysis, and visualization. It integrates with QC and trimming outputs, allowing for further exploration, and includes plugins for state-of-the-art sequence quality control like DADA2 and Deblur (Bolyen et al., 2019).

Handling large metagenomic datasets can strain resources, but incorporating QIIME2 with cloud platforms like Microsoft Azure, AWS, and Google Cloud poses a scalable solution (Callaghan, 2023). This approach allows researchers to efficiently handle and analyze large datasets, making advanced metagenomic analysis more accessible and manageable.

### 3.4 Taxonomic profiling

Taxonomic profiling, which predicts the relative abundance of taxa in metagenomic samples, involves read alignment, taxonomic classification, and abundance estimation (Lapierre et al., 2020). In AD, Greengenes and SILVA databases commonly assign taxonomy to sequences after the sequencing run and QC. Although it can be computationally demanding, SILVA is widely used due to its high-quality curation, frequent updates, and comprehensive coverage. Greengenes, while less frequently updated, is also commonly used, particularly for its focus on bacteria and archaea. Alignment maps sequencing reads to reference genomes to identify microbial taxa, annotate functional genes, and ensure data quality. However, incomplete reference databases can limit accuracy, though regular updates like those in the Genome Taxonomy Database (GTDB) help (Parks et al., 2022). The high computational demand of processing large datasets can be optimized using efficient tools like Bowtie2 and BWA (Robinson et al., 2017). Handling divergent sequences is challenging, so tools like HISAT2, Bowtie2, or hybrid approaches are recommended for better accuracy. Metaling, a novel method, balances precision and speed by using containment min hash (a technique that can rapidly estimate dataset similarities via k-mers hashing and identifying overlaps) for pre-filtering, reducing computational cost and enhancing profiling accuracy (Lapierre et al., 2020).

In the following alignment, contig assembly reconstructs microbial genomes to understand their roles in biogas production. Tools like SPAdes (effective for assembling short reads; Bankevich et al., 2012), MEGAHIT (fast and efficient for large datasets; Li et al., 2015), and Canu (designed for long-read data but primarily for single-genome assembly; Koren et al., 2017) are commonly used in AD. For complex metagenomic datasets typically in AD studies, long-read assemblers like Flye and HiCanu may be more suitable (Liu et al., 2023). Assembly merges overlapping sequences into contigs, which are essential for generating high-quality MAGs. Challenges include repetitive sequences, coverage gaps, and assembly errors. Hybrid assembly, combining short-read accuracy with long-read connectivity, improves accuracy and completeness. This method balances high-depth, short-read sequencing with lower-depth, long-read sequencing (Sims et al., 2014; Eisenhofer et al., 2024). Emerging tools like Unicycler and MaSurCA, applying short and long reads, help resolve repetitive sequences and reduce chimeric contigs (Zimin et al., 2013; Wick et al., 2017b; Liu et al., 2023). Notably, MaSuRCA corrects long reads with short reads before assembly, while SPAdes fix errors within the assembly graph, further enhancing accuracy (Zimin et al., 2013).

Contig binning groups of DNA fragments from the same or related genomes is essential for reconstructing MAGs and identifying metabolic pathways. Moreover, it can lead to the discovery of novel species and enzymes (Campanaro et al., 2018; Wang et al., 2024). Without binning, uncultured microbiomes in AD systems would remain unknown, leaving significant gaps in understanding their metabolic capabilities and potential for optimizing biogas production. Binning methods include sequence composition, abundance, and hybrid approaches, with tools like MetaBAT2 and CONCOCT known for high accuracy but requiring significant computational resources (Alneberg et al., 2014; Kang et al., 2019). Maxbin2 is effective with multiple metagenomic datasets and faces computational intensity challenges, employing an Expectation–Maximization algorithm to assign contigs based on tetranucleotide frequency and coverage (Wu et al., 2016). Advanced methods like COMEBin, using contrastive multi-view learning and data augmentation, efficiently recover MAGs, providing deeper perceptions into AD microbiomes (Wang et al., 2024).

Reconstructed MAGs must be checked for completeness, contamination, and errors. CheckM is widely used for assessing MAG quality, balancing accuracy and computational efficiency (Parks et al., 2015). GUNC, a novel tool that utilizes an entropy-based measure of lineage homogeneity across contigs, can infer each gene across different levels, using taxonomy to approximate phylogenetic relationships. GUNC identifies chimerism caused by horizontal gene transfer, improving MAG accuracy in complex environments. However, it is computationally intensive and requires comprehensive reference databases (Orakov et al., 2021). Combining tools such as GUNC, GTDB-Tk, and CheckM refines MAG datasets. Tools like GTDB-Tk and PhyloPhlAn4 provide high-resolution taxonomic classification but demand significant computational resources. GUNC can further refine results, leading to more precise outcomes.

### 3.5 Functionality profiling

Before initiating functional profiling, additional MAG refinement is recommended to ensure accuracy and avoid the misinterpretation of key metabolic pathways. Refining MAGs increases functional annotation accuracy, improving system interpretability. Tools such as RefineM, which are used alongside CheckM, can redefine boundaries, remove contaminants, and correct binning errors.

High-quality MAGs are ideal for accurate gene prediction. Prodigal is efficient for fast gene prediction but may struggle with fragmented or low-quality MAGs. Alternatives like FragGeneScan, Glimmer-MG, and MetaGeneMark (which employs hidden Markov models) handle sequencing errors and diverse data, while Prokka offers a streamlined annotation pipeline, though less flexible for non-bacterial genomes designed for gene prediction from short reads, effectively accounting for sequencing errors and codon usage (Taş et al., 2021; Yang et al., 2021). Artificial intelligence (AI) applications like Meta-MFDL and CNN-MGP improve accuracy but are computationally intensive and require well-annotated training data, which can be scarce in AD environments (Yang et al., 2021).

Functional microbiomes, interspecies interactions, and versatile metabolic pathways are critical for the microbial degradation processes in AD, which are essential to achieving efficient and targeted operations. Although studies traditionally focused on cultivated microbial members, recent advances in 16S rRNA gene amplicon sequencing, metagenomics, and other omics technologies (metatranscriptomics, metaproteomics, and metabolomics) have expanded knowledge of uncultivated microbes, microbial black holes, niche differentiation, and previously unknown metabolic pathways (Zhang X. et al., 2023), providing a more holistic view of MC function and activity in AD.

Whereas 16S rRNA amplicon sequencing data is conventionally not directly linked to functional potential, improved methods such as PICRUSt2 and Tax4Fun2 can predict microbial function from 16S rRNA, enhancing the ability to infer functional profiles from taxonomic data. However, they still depend on the quality and completeness of reference genomes (Douglas et al., 2020; Wemheuer et al., 2020). Additionally, combining 16S rRNA amplicon sequencing with MAGs has proven beneficial in uncovering abundant, previously undescribed lineages within key functional groups (Singleton et al., 2021), facilitating the identification of novel metabolic pathways and understanding ecological roles.

High-quality MAGs are necessary for exploring complex MCs. Databases like MiDAS connect microbial function with process data, while tools like Prokka and Infernal aid gene identification and annotation (Singleton et al., 2021). KEGG is widely used for mapping metabolic pathways, yet its focus on well-characterized organisms can be limiting. Integrating KEGG with databases like Eggnog and Pfam expands functional annotation with varying specificity (Huerta-Cepas et al., 2019; Palù et al., 2022). Pfam effectively identifies protein families and domains but may miss novel proteins, which can be addressed alongside *de novo* prediction tools (Coggill et al., 2008).

A recently developed cutting-edge software called METABOLIC is noteworthy for advancing microbial ecology and biogeochemistry studies. It integrates protein annotation from KEGG, TIGRfam, Pfam, custom hidden Markov model databases, dbCAN2, and MEROPS and assesses metabolic pathways through KEGG modules. METABOLIC profiles metabolic and biogeochemical traits and functional networks in MCs based on MAGs. METABOLIC provides protein annotations and metabolic pathway analyses for inferring the contribution of microorganisms, metabolism, interactions, activity, and biogeochemistry at the community scale. This software facilitates the standardization and integration of genome-informed metabolism into models, enabling easier interpretation of system metabolism and biogeochemistry (Zhou et al., 2022).

A feasible choice to enhance the quality and accuracy of genome-scale metabolic models is the amalgamation of MAG data with KEMET, a novel tool that expands KEGG annotations by identifying missing orthologs through hidden Markov models. However, KEMET may be limited by its reliance on high-quality reference genomes, affecting its performance in poorly characterized environments (Palù et al., 2022).

HUMAnN3, an innovative approach for functional profiling and metabolic pathways analysis that combines the MetaCyc database (Beghini et al., 2021; Cortés et al., 2022), can also be used for KEGG orthology enrichment analysis (Chen et al., 2021). A disadvantage is that it is computationally intense and requires extensive reference data.

Identifying metabolic pathways aids the prediction of metabolic and biochemical functional trait profiles across datasets, including MAGs, single-cell amplified genomes, or pure culture genomes (Taş et al., 2021). This knowledge is crucial for optimizing microbial processes in AD operations. Despite these advancements, challenges regarding better computational resources, improved functional annotation methods, and more comprehensive databases remain significant barriers in metagenomic workflow analysis.

### 3.6 Downstream analysis

In microbiome analysis, alpha and beta diversity metrics assess variation within and between MC. Alpha diversity is measured by species richness (Chao1) and evenness (Shannon index), with the latter being less affected by sequencing depth (Zhang et al., 2022). Rarefaction or normalization ensures accurate sample comparison, determining MC coverage and saturation despite sequencing depth variations (Stolze et al., 2015). Beta-diversity evaluates dissimilarity between MCs using metrics like Bray-Curtis and weighted UniFrac, or qualitative metrics, with ordination techniques like PCoA and PCA for visualization. QIIME, Mothur, and the R package vegan are suitable for computing alpha and beta diversity (Knight et al., 2018).

Multivariate analyses surpass simple correlations by applying sophisticated techniques to capture complex relationships between MCs and environmental variables. Symmetric methods, like canonical correlation analysis, co-inertia analysis, and Procrustes analysis, treat variables equally without distinguishing between explanatory or response roles. Conversely, asymmetric approaches, like redundancy analysis and canonical correspondence analysis, differentiate between explanatory and response variables. Non-metric multidimensional scaling is an exploratory method mainly for visualizing sample similarities or dissimilarities. Methods like generalized linear models and permutation tests assess the significance of multivariate patterns. These analyses rely on detailed metadata, high-dimensional data, and well-structured experiments (Ramette, 2007; Paliy and Shankar, 2016).

Recent methods combine sequencing with microbial cell counts for accurate data, enabling ML approaches like random forest regression to distinguish samples based on metadata. SourceTracker, a Bayesian tool, identifies microbial sources and classifies samples. However, ML analysis demands large datasets and cross-validation to ensure robustness (Knight et al., 2018).

Analyzing alpha- and beta-diversity in AD studies is decisive for understanding MC structure and stability, impacting biogas production. While trained ML models improve data analysis efficiency, they require substantial training data and may struggle with adaptation, highlighting the need for more flexible models with less dependency on extensive data inputs.

### 3.7 Metagenomics resources used within the AD research field

Selecting appropriate tools for metagenomic analysis is vital, and researchers must match their selections to the specific requirements and resources of their projects. Comprehensive platforms such as MOTHUR, QIIME2, MG-RAST, and MEGAN can manage most of the metagenomic workflow, making them suitable for budgets with limited budgets or simple goals. Moreover, tools such as MOCAT2 and MetaWRAP efficiently cover processes from gene prediction to functional profiling (Taş et al., 2021).

However, these all-in-one solutions may lack the precision and adaptability of more specialized tools, such as SPAdes for genome assembly, Kraken2 for taxonomic classification, and MetaPhlAn for strain-level profiling. Advanced tools such as KEMET, METABOLIC, PICRUSt2, CNN-MPG, and GUNC provide greater accuracy and functionality but require significant computational resources and expertise. The future of metagenomic research may involve the integration of specialized tools into more user-friendly platforms that offer both precision with ease of use.

Table 4 presents a range of methods and tools, both common and novel, used for metagenomic analysis in AD research, spanning from sequencing type selection to functional analysis.

**Table 4:** Process and tools used during metagenomic analysis within the AD research field.

Sequencing length	Sequencing type	Sequencing system	Region	Sequencing library preparation	Data cleaning and assessment	Taxonomy	Function annotation	Citation
Short	16S rRNA gene High-throughput sequencing	Illumina MiSeq	V3	Quick-16S NGS Library Preparation	DADA2	Greengenes, QIIME1.9.1		Akyol et al. (2019)
			V3-V4	Illumina MiSeq	MOTHUR	SILVA, RDP Classifier	KEGG	Bedoya et al. (2019)
				Nextera XT	Trimmomatic/MOTHUR/QIIMME	BLAST2.2.22/RDP Classifier	-	Khanthong et al. (2023)
					QIIME2/DADA2	SILVA, DADA2’s q2-feature-classifier	-	Cheng et al. (2024)
				NGS library	QIIME1.9.1	SILVA, QIIME	-	Murillo-Roos et al. (2022)
				-	QIIME1.17	RDP Classifier	PICRUSt/FUNGuild	Zhao et al. (2023)
				-	DADA2	GreenGenes	PICRUSt/KEGG	Pei et al. (2022)
			V4	-	QIIME2/DADA2	SILVA QIIME	PICRUSt2/KEGG	Trego et al. (2021)
				Illumina MiSeq	FastQC/USEARCH	QIIME1.9.1/Uclust Consensus Taxonomy Assigner/*de novo* taxonomy	-	Vendruscolo et al. (2020)
			V4-V5		QIIME	SILVA, UPARSE	-	Chen et al. (2024)
					MOTHUR	MOTHUR	-	Venkiteshwaran et al. (2017)
	16S rRNA gene pyrosequencing	Ion PGM System		Ion PGM Hi-Q	Ion Reporter	Greengenes, Ion Reporter	-	Han et al. (2017)
		Illumina HiSeq 2000	V3-V4	Illumina HiSeq 2000	MOTHUR	MOTHUR	KEGG	Zhang et al. (2024)
	Shotgun sequencing	Illumina Novaseq 6,000	Whole genome	Nextera DNA Flex	Trimmomatic	GTDB-Tk1.7.0	Prodigal2.6.2/METABOLIC/KEGG	Greses et al. (2023)
		NextSeq 500		Nextera XT	Trimmomatic	PhyloPhIAn/CheckM	Prodigal2.6.2/GhostKOALA-KEGG/EggNOG/MAPLE2.3	Fontana et al. (2018)
	Pyrosequencing	Roche 454 FLX			In-house script	MEGAN	MG-RAST	Bedoya et al. (2019)
Long	Oxford nanopore sequencing	MinION sequencer	Whole genome	LSK-108 LSK-109	Nanoplot	Centrifuge		Brandt et al. (2020)
Short/Long	Hybrid (Shotgun sequencing/Oxford nanopore sequencing)	Illumina NextSeq 500/Oxford Nanopore MinION sequencer	Whole genome	Nextera DNA Flex/SQK rapid sequencing	Trimmomatic	GTDB-Tk1.3.0	Prodigal2.6.2/EggNOG2.0.1/Diamond/RAST/KEMET	De Bernardini et al. (2022)

From Table 4, it is evident AD researchers predominantly favor short-read approaches targeting the V3, V4, and V5 regions, likely due to project scope, practical experience, or budget constraints. Short-read sequencing, often paired with Illumina NGS or Nextera XT kits, is cost-effective and scalable, though it may miss novel genomes. In contrast, while more expensive, long-read sequencing delivers high-fidelity genome recovery and deeper microbial insights. Hybrid approaches, which combine the strengths of both methods, balance cost with comprehensive analysis.

Researchers should select sequencing strategies based on specific research needs, whether focusing on 16S rRNA gene regions for targeted studies or whole-genome sequencing for broader analysis. There is a growing interest in AD research in correlating MC, functional annotation, and metabolic pathway identification, aiming to improve biogas yields and produce valuable products such as biomethane, hydromethane, and hydrogen, along with byproducts like organic acids, alcohols, and biofertilizers.

Metagenomics and hybrid sequencing are increasingly preferred for exposing the microbial “black box” of AD systems, which is essential for enhancing biogas production. Tools like MOTHUR for versatile analysis stage and KEGG for functional annotation are popular in AD research. As costs decrease and technologies advance, integrating high-fidelity sequencing with robust bioinformatics is expected to enhance AD process understanding and efficiency. Additionally, metagenomics, along with other omics technologies, has proven effective for uncovering key functional associations and tracking gene expression, demonstrating the power of multi-omics in advancing AD research.

## 4 Applications of metagenomics in anaerobic digestion research

Operational factors in AD, such as the feeding regime (mono and co-digestion), temperature, reactor type, pH value, substrate choice, organic loading rate (OLR), and hydraulic retention time, can influence MC structure, dynamics, and interactions (Su et al., 2019; Pasalari et al., 2021). Understanding these variations is crucial for informed decisions that lead to more efficient AD systems, a task that is now more feasible thanks to metagenomics analysis.

Among factors influencing AD, the choice of substrate is crucial, as it significantly influences MC. Studies have shown a strong link between changes in MC and the substrate used in both mono-digestion and co-digestion processes. The composition of the feedstock plays a major role in shaping the active microbiota within AD systems (Tsapekos et al., 2017; Zhang et al., 2020a).

Furthermore, concerning reactor configurations, two of the most common setups in biogas are single-stage and two-stage continuous stirred tank reactors. Through metagenomic analysis, different microorganisms and their syntrophic relationships within a reactor can be unraveled, aiding in understanding how these factors can influence community structure, interactions, and overall process efficiency (Treu et al., 2016). For instance, using dairy wastes in a two-stage reactor, (Fontana et al., 2018) identified that certain microorganisms were predominant in the first (acidogenic) reactor, while others were more active in the second (methanogenic) reactor. This niche differentiation through metagenomics demonstrated that a two-stage reactor could benefit methane production and how MC can influence the AD process.

Moreover, temperature also plays a significant role in shaping microbial ecology. When comparing mesophilic and thermophilic biogas plants, the use of Genome Centric Metagenomics (GCM) was crucial because it allowed a more detailed examination of communities at the species level, providing insights into specific roles of individual microorganisms in a complex MC (Campanaro et al., 2018). In this study, thermophilic microorganisms were more efficient in breaking down complex organic compounds into methane than mesophilic ones. The Genome Centric Metagenomic (GCM) approach allowed us to understand how these temperature-dependent microorganisms contribute to the overall efficiency and stability of the AD process.

Previous examples demonstrate that the application of metagenomics in AD research has significantly enhanced the understanding of MC and their functional roles in the AD process. By providing exhaustive knowledge into the abundance, diversity, and interactions of microorganisms, as well as identifying keystone species and syntrophic relationships, metagenomics has paved the way for improved analysis and decision-making in AD systems. Likewise, evaluating the impact of varied inoculums on long-term anaerobic yield (Peces et al., 2018) suggested that achieving a stable process requires establishing and maintaining a desirable MC composition through careful planning and optimization of operational conditions. This knowledge can be leveraged to optimize process conditions and enhance microbial activity to increase biogas yield and process efficiency. Thus, in the following sections, the microbiota composition in relation to different substrates in the two feeding regimes, mono- and co-digestion in AD, will be explored.

### 4.1 Microbial community composition during mono-digestion

Understanding the MC is fundamental for optimizing AD. In mono-digestion, a single type of substrate is used as the sole feedstock for biogas production. Some common single substrates include pig manure, rice straw, corn stalk, or silage. These last two are differentiated, with the first consisting of plant residues that remain after harvest and silage referring to the fermented forage from the entire plant.

The substrate choice, carbon-to-nitrogen ratio (C/N), and total solids content are crucial factors affecting the OLR. High total solids content may require inconveniently large water additions, while a low C/N ratio might necessitate adjusted feeding strategies to avoid digester overload and process imbalances due to acidification or VFA accumulation (Pore et al., 2016). A feeding regime is essential to maintain a stable MC, ensuring they have sufficient time to adapt and function effectively.

**Figure 2:**
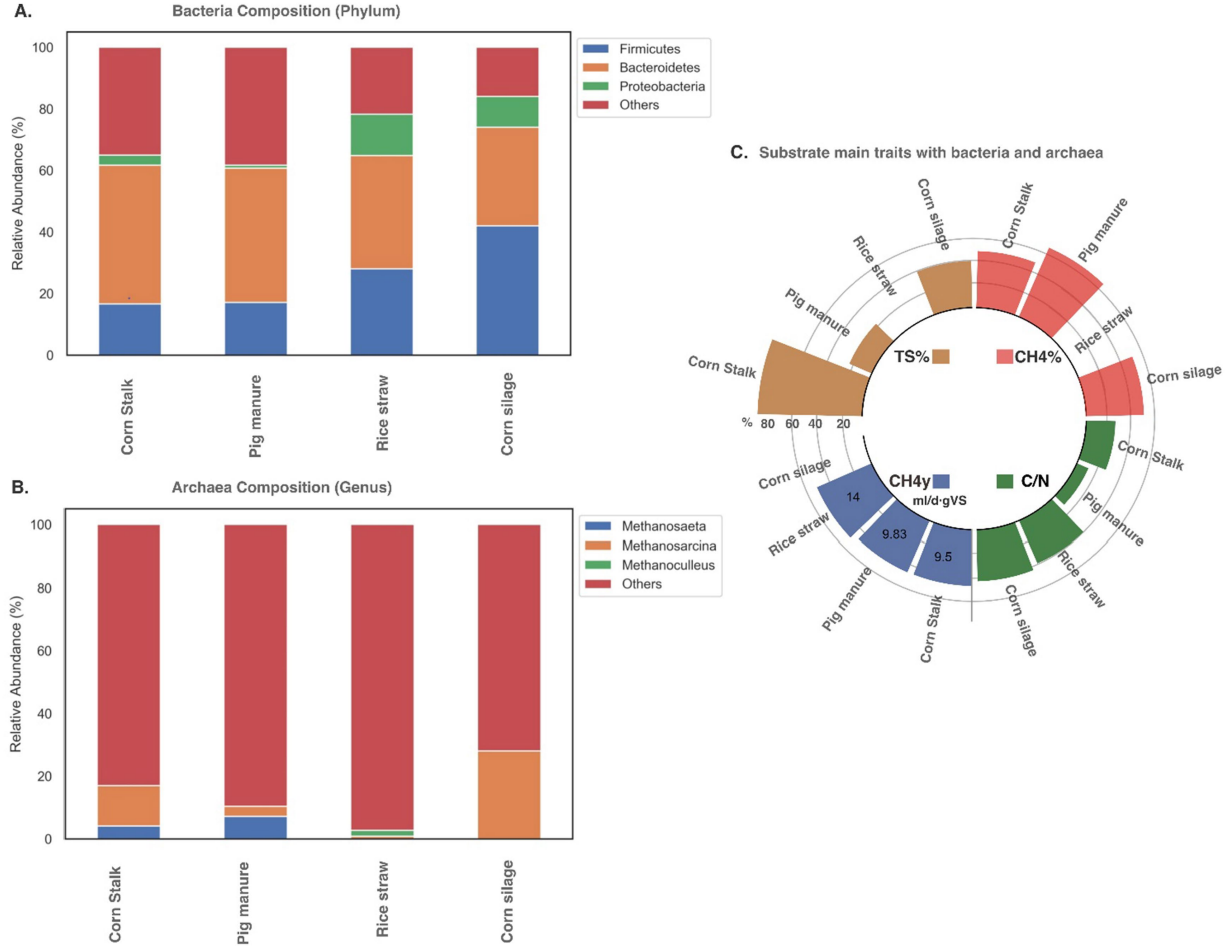
Microbial community composition during monodigestion: (A) bacteria and (B) Archaea composition and (C) substrate characteristics in terms of total solids (TS%), methane content (CH_4_), methane yield (CH_4y_) and carbon/nitrogen ratio (C/N) described in mesophilic digesters using average levels from different studies exemplifying corn stalk (Wang et al., 2017), pig manure (Wang et al., 2017), rice straw (Pore et al., 2016), and corn silage (Wirth et al., 2015).

An analysis was conducted using data from different studies under mesophilic conditions to understand the relative abundance of the MC using different substrates and their relative C/N ratio, TS%, CH_4_% content, and CH_4_ yield. Each study focused on a different substrate and described the MC abundance through a 16S rRNA gene metataxonomic, taking a sample once the system was stable. The resulting figures highlight the most common phyla (Figures 2A,B) and the relation to each substrate’s TS% and C/N ratio (Figure 2C).

This analysis reveals that *Firmicutes*, *Bacteroidetes*, and *Proteobacteria* are the most abundant bacteria across the four analyzed substrates (Figure 2A). *Bacteroidetes*, the dominant phylum, account for 23–36% of the MC and play a key role in breaking down cellulose and hemicellulose during acidogenesis into heteropolysaccharides like glucose and D-xylan, essential for lignocellulose degradation (Wirth et al., 2015; Heyer et al., 2016; Jia et al., 2016). Common hemicellulose-degrading species include *Bacterioides fibrisolvens*, *Bacterioides ruminicola*, *Ruminococcus albus*, and *Ruminococcus flavenfaciens* (Maspolim et al., 2015). *Streptomyces* is notable for its metabolite production and lignocellulose degradation, and it is frequently highlighted (de Lima Procópio et al., 2012; Zhang et al., 2019a). The consistent abundance of *Bacteroidetes* (Figure 2C), despite varying TS% and C/N ratios, highlights their adaptability and critical role in AD processes.

The second most abundant phylum, *Firmicutes*, involved in cellulose degradation (Wirth et al., 2015), accounted for 42% relative abundance in maize silage, with lower levels observed in corn stalk and pig manure. High TS% is believed to promote both *Bacteroidetes* and *Firmicutes*, though the lower C/N ratio in corn stalk compared to silage may have affected the abundance of *Firmicutes*.

The elevated TS% in corn stalks (Figure 2C) delays the initiation of microbial activity. Therefore, during hydrolysis and acidogenesis, key degradative bacteria from the *Firmicutes* and *Bacteroidetes* phyla can reach abundances of 60% (Pore et al., 2016; Wang et al., 2017) to 90% (Liu et al., 2019). Firmicutes is considered a versatile phylum capable of degrading lipids, carbohydrates, and proteins, reflecting the digester’s ability to metabolize cellulose, lignin, proteins, and sugars, which highlights the importance of this phylum in AD.

Furthermore, *Proteobacteria* were present to a lesser extent, with a relative abundance between 2 and 17% across samples (Figure 2A). Reduced *Proteobacteria* could be attributed to the high water content in materials like pig manure, which is associated with low TS%. This condition is believed to promote the proliferation of *Bacteroidetes* and *Firmicutes* while diminishing the population size of *Proteobacteria* (Xu et al., 2018b). Furthermore, the C/N ratio in pig manure is low compared to the optimal C/N value of 20–30 (Wang Y. et al., 2018; Xu et al., 2018b). This characteristic of pig manure as a single substrate is likely a limiting factor for microbial activity.

Archaea, single-celled prokaryotes, are found in a smaller proportion compared to bacteria, with ratios varying from 50 to 1,000 (Wang Y. et al., 2018) to 34.2 to 67.78% (Heyer et al., 2016). Some authors attribute these variations to methodological bias (Heyer et al., 2016). Despite their lower numbers, they are crucial in AD, serving as dominant methanogens, though many archaea contributing to methanogenesis remain unidentified (Bedoya et al., 2019; Pore et al., 2016). This study found many archaea that had not yet been described (Figure 2B). However, organisms such as *Methanosarcina* and *Methanosaeta*, known to play key roles in methanogenesis, were identified (Pore et al., 2016; Wang et al., 2017).

*Methanoculleus*, a hydrogenotrophic methanogen, was identified in rice straw as vital for biogas production (Xu et al., 2018b). In corn silage, *Methanosarcina* likely performed the entire methanogenesis phase, including hydrogenotrophic, acetoclastic, and methylotrophic reactions (Wang et al., 2017), while *Methanothrix* is related to methane production from acetate (Wirth et al., 2015).

In mono-digestion, pig manure exhibited the highest CH_4_% content, while rice straw achieved the highest CH_4_ yield. *Bacteroidetes* likely played an important role in degrading cellulose and hemicellulose in pig manure and corn stalks (Ren et al., 2014). The shared preference for acetate between *Firmicutes* and *Methanosarcina* may have enhanced acetoclastic methanogenesis (Xu et al., 2018b). The C/N ratio in single-substrate digestion is crucial, as imbalanced ratios can disrupt digester operation and diminish buffering capacity. Since single monodigestion substrates are rich in either carbon or nitrogen, careful selection of raw materials is essential to maintain a balanced C/N ratio, ensuring stable MC and system performance (Wirth et al., 2015; Wang P. et al., 2018).

### 4.2 Microbial community composition and associated syntrophic during codigestion

**Figure 3:**
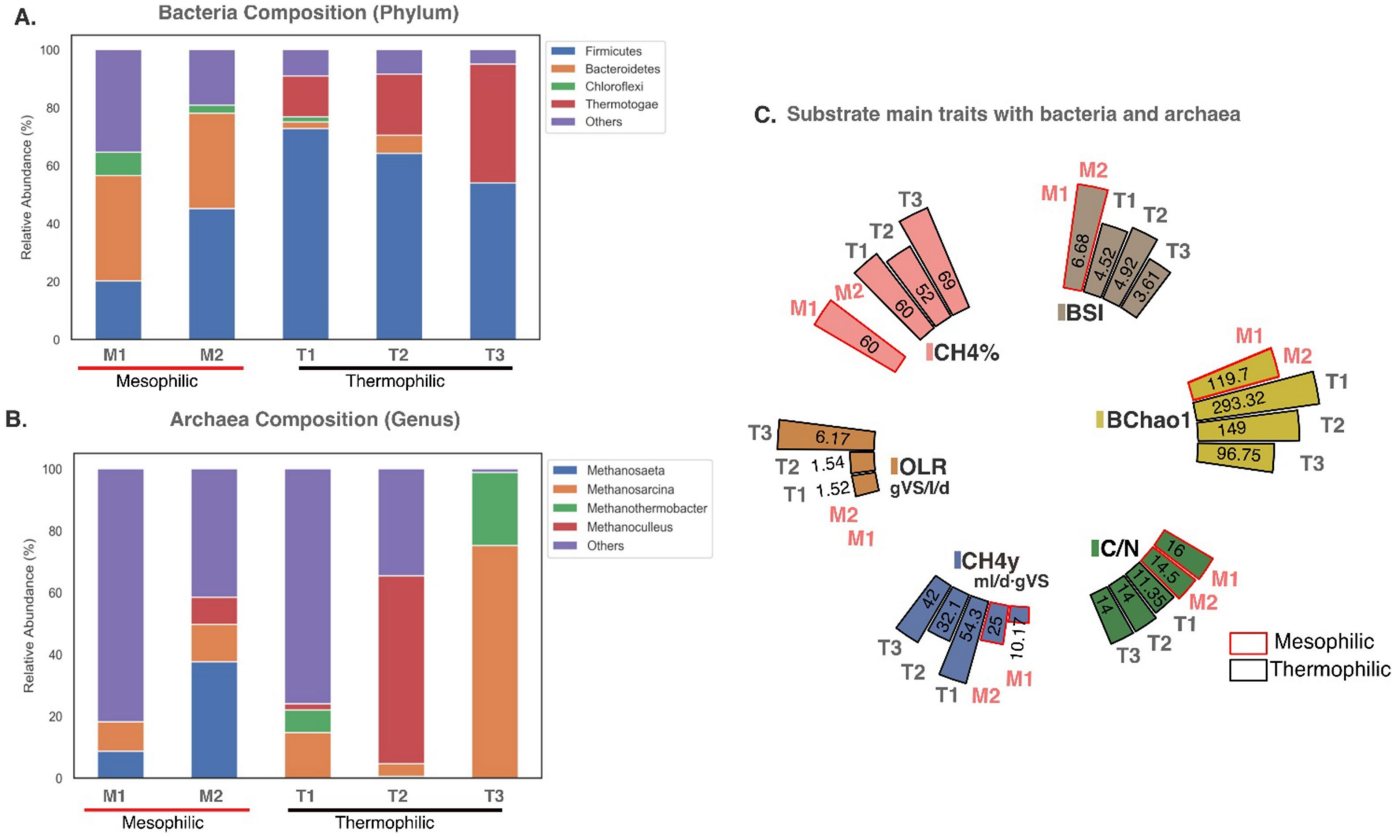
Microbial community composition during codigestion: (A) Bacteria and (B) Archaea composition described in mesophilic (M) and thermophilic digesters (T), M1 and M2 correspond to mesophilic digesters with one chamber configuration, M1 with substrates such as pig manure, corn stalk and activated sludge (Wang et al., 2017) and M2 with pig manure, corn stalk and cucumber residue (Wang Y. et al., 2018). In thermophilic digesters, T1 had a one-chamber configuration with substrates of food waste, activated sludge and biochar treatment (Zhang et al., 2020b), T2 had a two-chamber configuration with substrates like food waste, horticultural waste and activated sludge (Zhang et al., 2020a); and T3 had a three-chamber configuration with substrates as food waste, horticultural waste and activated sludge (Zhang et al., 2020a). (C) Represent main characteristics of the five treatments described above in terms of diversity: BSI (Diversity index Shannon-Wiener for bacteria), BChao1 (richness index for bacteria Chao1), substrate trait such as C/N ratio, OLR (organic loading rate) and associated CH_4_% (methane concentration) and CH_4_y (CH_4_ yield).

Co-digestion involves the simultaneous anaerobic digestion of two or more substrates, often developed to optimize biogas production and improve the stability of the digestion process. In this study, a comparative analysis of the MC in the codigestion system was analyzed by comparing two mesophilic digesters and three thermophilic digesters, each with different substrates. The mesophilic systems used a co-digestion base mixture of pig manure and corn stalk, with one system incorporating activated sludge as inoculum and the other adding cucumber residue. The thermophilic systems used a base mixture of food waste and activated sludge: one with a two-chamber configuration, another with a three-chamber configuration, with two adding horticultural waste, and a third with a single-chamber setup with the addition of biochar as a conductive material. Data from each digester was sourced from different studies, with samples taken for metagenomic analysis after system stabilization (Figure 3).

The phyla *Bacteroidetes* and *Firmicutes*, dominant acid-forming bacteria, were prevalent in mesophilic digesters. *Chloroflexi*, an acetic acid producer capable of degrading polysaccharides and monosaccharides (Wang et al., 2017), was prominent in mesophilic systems without cucumber residue and less abundant in thermophilic systems (Figure 3A). *Bacteroidetes*, known for strong adhesion to starch particles (Ren et al., 2014), were abundant in mesophilic digesters, likely facilitating the production of VFA, CO_2_, and H_2_ (Murillo-Roos et al., 2022). For mesophilic treatment (Wang Y. et al., 2018), pure cucumber residue led to acidification and low methane production during mono-digestion. However, a 5:2:3 mixture of pig manure, corn stalks, and cucumber residue improved microbial diversity and methane production, highlighting the benefits of co-digestion (Figure 3C).

*Firmicutes* were abundant in both mesophilic and thermophilic treatments, with a particularly high abundance in thermophilic systems. In thermophilic systems, *Thermotogae* was commonly observed, with its abundance increasing as the number of digestion stages grew. It is believed that *Thermotogae*, through syntrophic degradation of acetate with hydrogenotrophic methanogens, thrives in multi-stage digesters where acidogenesis and methanogenesis are separated. This separation allows for the proliferation of more specialized methanogenic bacteria (Xu et al., 2018b). This syntrophic relationship between *Thermotogae* and methanogens likely contributed to the higher methane content and yield observed in the three-chamber configuration system.

The use of biochar in thermophilic systems improved microbial diversity, methane content, and yield, demonstrating its potential to stabilize systems by promoting direct interspecies electron transfer (DIET) to accelerate microbial metabolism (Gahlot et al., 2021; Johnravindar et al., 2021; Su et al., 2023), and mitigate high OLR impacts (Ye et al., 2018; Zhang et al., 2020b). Biochar treatments yielded the highest CH_4_ at the lowest OLR (1.52gVS/l/d) and showed the greatest BSI and BChao1 (Wang Y. et al., 2018; Zhang et al., 2020b), indicating greater microbial diversity and richness (Xu et al., 2018a; Zhang et al., 2020a, 2020b).

Figure 3B shows that codigestion increased the abundance of dominant archaea and introduced new participants, such as *Methanothermobacter*, in thermophilic systems and *Methanoculleus* in mesophilic and thermophilic treatments. No single dominant archaea phylum was present across all digesters, though *Methanosarcina* appeared in all treatments, particularly dominating the three-chamber thermophilic system. This dominance may be due to its ability to utilize diverse substrates like acetate, methanol, and H_2_ and thrive across a wide temperature range (Ali Shah et al., 2014; Murillo-Roos et al., 2022).

*Methanothrix* dominated over *Methanosarcina* in mesophilic digesters, with the latter absent in thermophilic treatments. *Methanothrix* likely prevailed due to its higher acetate affinity, effectively lowering acetate concentration compared to *Methanosarcina* (Xu et al., 2018b; Oosterkamp et al., 2019). *Methanoculleus* and *Methanothermobacter* were identified in thermophilic digesters at different OLRs (Figure 3C), with low OLR favoring *Methanoculleus* in the two-chamber system, while high OLR advantaged *Methanothermobacter* in three-chamber systems (Zhang et al., 2020a). Both *Methanothermobacter* and *Methanosarcina*, key hydrogenotrophic microorganisms, were identified (Bedoya et al., 2019).

Phylum *Chloroflexi* such as *Anaerolineae* class is related to hydrogenotrophic methanogens (Xu et al., 2018b), Archaea *Methanosaeta* spp. (Bovio-Winkler et al., 2021), and acetoclastic methanogens like *Methanosarcinaceae* (Liang et al., 2015; Murillo-Roos et al., 2022), where *Anaerolineae* is an acetate or hydrogen contributor to methanogens (Bovio-Winkler et al., 2021; Murillo-Roos et al., 2022). This potential syntrophy could promote a flow of metabolites between the three, leading to higher methane yield.

Since syntrophy activity is an important aspect of AD system stoutness (Oosterkamp et al., 2019), Figure 4 displays common trophic microbial associations, identified through metagenomic studies, that have assisted biogas production.

**Figure 4:**
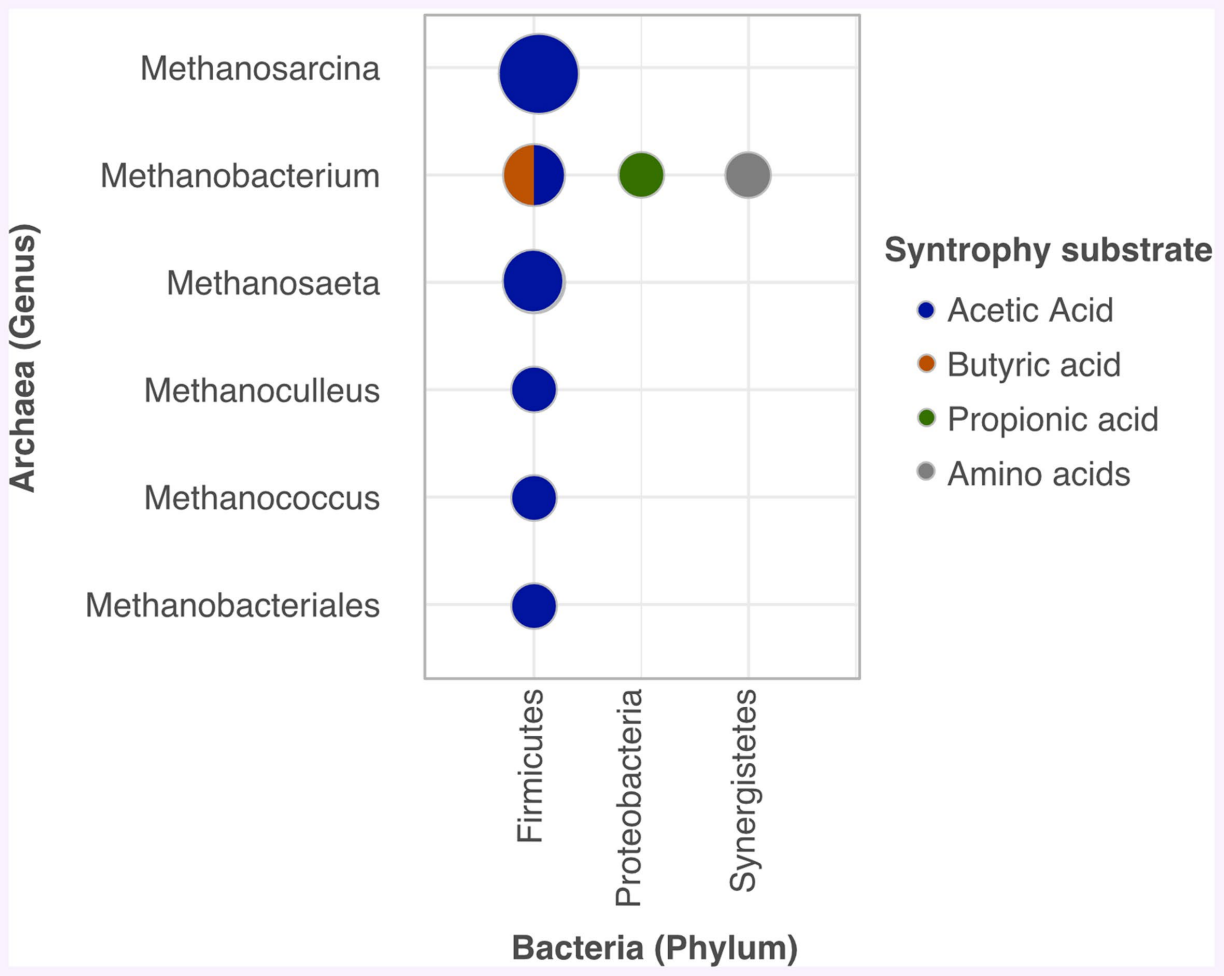
Common syntrophies in AD.

To reiterate, *Firmicutes* stands out as a key player, linked to all archaea organisms Figure 4. It co-cultures acetate with *Methanobacteriales*, *Methanobacterium*, *Methanococcus*, *Methanoculleus*, *Methanothrix*, or Methanosarcina (Saha et al., 2019; Han et al., 2020; Tabatabaei et al., 2020); and butyrate with *Methanobacterium* to produce propionic acid, which is converted into biogas (Han et al., 2020). As shown in Figure 4, *Proteobacteria* also contribute to biogas production from propionic acid when associated with hydrogenotrophic methanogens such as *Methanobacterium* (Han et al., 2020). Similarly, syntrophy between *Aminivibrio* (*Synergistetes*) and *Methanobacterium* facilitated methanation via VFA from amino acid oxidation (Saha et al., 2019). *Thermotogae* was also observed to degrade acetate with hydrogenotrophic methanogens (Xu et al., 2018b).

In summary, the treatments shown in Figure 3 had an average C/N ratio of 14, which yielded optimal results, suggesting this to be a suitable C/N ratio for enhancing microbial biomass digestibility (Wirth et al., 2015). Codigestion consistently proved to be a more cost-effective method than monodigestion, offering improvements in buffering capacity, nutrient equilibrium, and overall process stabilization (Wang et al., 2017; Wang Y. et al., 2018). Achieving a balance of macronutrients and micronutrients while reducing inhibitory and toxic compounds, further supports system efficiency (Wang Y. et al., 2018). Then, it could be stated that codigestion induces the creation of favorable microenvironments that promote methanogen growth and system stabilization, leading to increased CH_4y_ yield. These microenvironments are occupied by keystone microbial members, considered AD regulation markers, which are responsible for sustaining the AD process (Xu et al., 2018a). The absence of these key organisms could lead to AD failure.

Acidogenic bacteria from the *Firmicutes* phylum play a fundamental role in AD as a dominant, versatile, and functionally diverse organism (Figures 3, 4), capable of metabolizing complex organic residues like lipids, carbohydrates, cellulose, lignin, proteins, and sugars under various conditions (Ren et al., 2014; Venkiteshwaran et al., 2017; Zhao et al., 2018). *Firmicutes* are positively connected to biogas production (Xu et al., 2018b). *Bacteroidetes*, the second most dominant acidogenic bacteria, also play a significant role in AD (Wang P. et al., 2018). Both phyla are key in degrading substrates, producing acetic acid, and secreting lytic enzymes during acidogenesis, increasing soluble organic matter concentrations and CH_4y_ yield (Liu et al., 2019).

Acetate concentration influences acetotrophic methanogens regarding growth, CH_4_ production, and metabolism. For instance, *Methanothrix* can grow and generate CH_4_ at low acetate concentrations. *Firmicutes* also appear to have a syntrophic relationship with *Methanothrix* and hydrogenotrophic methanogen *Methanosarcina,* where an increase in *Methanosarcina* correlates with higher acetate levels, potentially stabilizing the system and enhancing CH_4_ generation (Xu et al., 2018b; Zhou et al., 2023).

The three main methanogenesis pathways present in AD are acetoclastic, hydrogenotrophic, and methylotrophic (Ali Shah et al., 2014). *Methanothrix*, an acetoclastic methanogen, efficiently converts low-concentration acetate into CH_4_ and CO_2_, while *Methanosarcina* can generate CH_4_ through all three pathways, making it a cornerstone organism in co-digestion (Wang et al., 2017; Oosterkamp et al., 2019). Methanothrix dominated mesophilic treatments but decreased notably in thermophilic treatments, where *Thermotogae* and *Methanothermobacter* became more prevalent. *Methanosarcina* was present in both treatments.

Inoculum material like activated sludge is vital for system startup and stability, balancing populations, and promoting syntrophic metabolism (Ali Shah et al., 2014). Bioaugmentation with H_2_ producers has shown that it is a bottleneck, with its reduction leading to increased biogas production (Heyer et al., 2016). Moreover, methods such as DIET and conductive materials like biochar improve methanogenic bioreactor performance. With its functional groups acting as electron shuttles and high electrical conductivity, biochar is favored for DIET (Ye et al., 2018; Alghashm et al., 2023). Alternative conductive materials like magnetite boost AD performance by adding *in situ* H_2_S removal and elemental sulfur recovery. Metagenomics combined with DIET has uncovered new electric syntrophies between sulfide-oxidizing bacteria (SOB) and electrotrophic *Methanothrix* and new routes for anaerobic sulfur metabolism, stimulating electroactive microorganisms (Jung et al., 2023; Zhou et al., 2023). Syntrophic collaboration between hydrogenotrophic methanogens and acetogenic bacteria, known as interspecies hydrogen transfer, maintains H_2_ levels low enough to make secondary fermentation thermodynamically feasible, harmonizing H_2_ production and consumption in anaerobic reactors (Ali Shah et al., 2014; Gahlot et al., 2021).

## 5 Advanced metagenomic analyses

### 5.1 Genome-centric metagenomics in AD research

**Figure 5:**
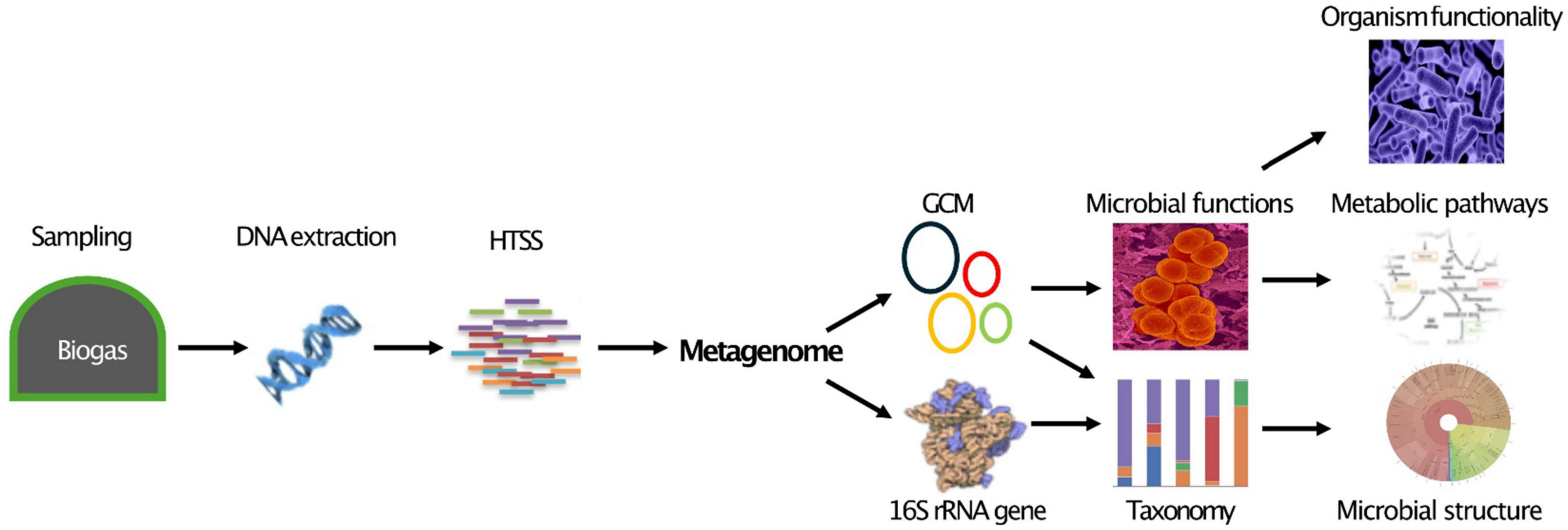
Process overview of metagenomic analysis including GCM enhancement and scopes.

16S rRNA gene sequencing provides a broad overview of MC during AD but may lack the depth needed to interpret functional and metabolic interactions crucial to process efficiency (Asgarineshat et al., 2022). GCM tools have demonstrated significant advantages over PCR techniques for enhanced analysis of microbiota metagenomes without PCR bias, augments coverage of high-GC regions of the genome, reduces duplicate reads (Sims et al., 2014), and mitigates inaccuracies in the abundance and diversity of genes (Kanagawa, 2003). GCM is a rapidly advancing method that can recover high-quality MAGs through efficient binning procedures (Treu et al., 2018; Jiang et al., 2023). This technique facilitates the elucidation of key microbial players, their functions, shared metabolic pathways, interspecies hydrogen transfer, cofactor identification, nutrient competition, nutrient exchanges, and the metabolic reconstruction of MAGs (Figure 5) (Campanaro et al., 2019; Zhu et al., 2019; Sun M. et al., 2023; Zhang L. et al., 2023). As a result, more accurate predictive models can be developed, allowing better correlations between microbial physiology to ecological fitness, organic material selection, digester design, and operation to achieve higher performance and biogas generation (Turaev and Rattei, 2016; Palù et al., 2022; Greses et al., 2023; Heyer et al., 2024).

Recognizing the importance of compositional structures and functional connections in AD, recent studies have characterized and compared these aspects under different feeding treatments (continuous and discontinuous) and OLRs (high and low), using short-chain fatty acids such as acetate, propionate, and butyrate as the carbon sources. To address the limitations of existing technologies in analyzing complex MCs, a hybrid approach combining Illumina-based short-read and ONP long-read assemblies can be a feasible solution. This methodology is expected to enhance MAG completeness and proximity, leading to the discovery of potentially novel organisms such as *Syntrophobacteraceae* species, associated with syntrophic short-chain fatty acid oxidation; *Syntrophomonadaceae* species, implicated in butyrate oxidation; and *Methanoculleus* species, linked to hydrogenotrophic methanogenesis (Becker et al., 2023).

Additionally, it has been learned that MC responsible for fundamental processes may have a broader role in the food web, potentially leading to the discovery of unknown metabolite cross-feeding interactions and new participants like scavengers in biomass turnover. For instance, *Syntrophobacter* and *Pelotomaculum* have been identified as syntrophic oxidants for propionate, while pathway analysis might reveal common butyrate degradation gene patterns, probably caused by novel butyrate-degrading bacteria like *Syntrophomonas*. In the oxidation of propionic and butyric acid, *Cloacimonetes*, *Cryptanaerobacter*, and *Desulfovibrio* can be identified. Unclassified *Bacteroidetes*, *Candidatus Cloacimonas*, *Mesotoga*, *Desulfovibrio*, or *Endomicrobium* may not exhibit significant abundance unless butyrate degradation is completed. *Desulfotomaculum*, which utilizes acetate, butyrate, and propionate oxidation, can be considered a metabolically versatile genus.

Similarly, hydrogenotrophic and acetoclastic methanogenesis pathway genes identified in the archaeal genus *Methanothrix* are believed to generate CH_4_ through DIET (Zhang X. et al., 2023). *Methanosarcina* has been identified as a resilient and abundant archaeon, capable of overtaking *Methanothrix* at high OLRs. This indicates that stress tolerance can be alleviated if *Methanosarcina* is promoted in exchange for more vulnerable species, boosting system resilience during AD. The increase in *methanosarcina* abundance through discontinuous regimes is interesting, advocating that feeding regimes might influence temporal niches and provide optimal conditions for archaeal community motion. This type of study emphasizes the significance of syntrophic activity between bacteria and methanogenic archaea, which could enhance system buffering and stability.

Innovative research by Gaspari et al. (2024) used GCM to assess the impact of rising ammonia levels on the resilience and functional dynamics of AD systems, with a focus on microbial responses to ammonia stress, particularly key archaeal species and methanogenesis pathways. Ammonia is a well-known inhibitor of biogas production, with concentrations of approximately 3,000 mg/L, depending on pH levels, potentially affecting AD (Bi et al., 2021; Gaspari et al., 2024).

Concentrations above this threshold, irrespective of pH, significantly inhibit microbial activity and biogas production (Kalamaras et al., 2023; Gaspari et al., 2024). Gaspari et al. (2024) revealed that under high ammonia conditions, hydrogenotrophic methanogenesis pathways were favored over the acetoclastic pathway. Dominant species identified included *Methanoculleus bourgensis MX4*, *Methanoculleus* sp. *MA7*, *Methano-thrix* sp. *MA6*, and *Methanosarcina flavescens MX5.*

Furthermore, the findings suggest that certain archaeal species, such as *M. flavescens MX5* and *Methanothrix* sp., *MA6* may adapt their metabolic strategies to survive and continue methane production via CO_2_ reduction, potentially utilizing DIET, indicating ecological fitness. The presence of syntrophic acetate-oxidizing bacteria (SAOB), such as *Syntrophomonadaceae* sp. *MX66,* likely contributed to increased hydrogenotrophic activity by producing H_2_ and CO_2_ from acetate, which could then be reduced via the acetyl-CoA pathway to sustain methane production (Becker et al., 2023; Gaspari et al., 2024).

This research highlights the importance of expanding metagenomics approaches to achieve deep insights into microbial dynamics and their response to environmental stressors. Advanced metagenomics techniques, like GCM, allow detailed analysis recognizing microbial interactions and metabolic pathways, providing a valuable foundation for informed decision-making in AD process optimization. For example, understanding the MC’s tolerance to specific stress factors like ammonia can guide the selection of suitable feedstock compositions. Additionally, by modifying procedure parameters like pH and temperature based on metagenomic insights, it becomes possible to enhance biogas production and identify potential diagnostic biomarkers for monitoring and controlling AD.

In a significant step forward for AD research, Greses et al. (2023) demonstrated the power of GCM in deciphering the intricate microbial dynamics of open-mixed cultures for producing value-added biochemicals. By focusing on functional metabolic pathways, the study assessed the potential of food waste as a sustainable feedstock for generating these valuable compounds, with the aim of replacing traditional petrochemical-based products. The researchers employed an in-silico community-level simulation to elucidate single-species activities and essential interspecies interactions within the culture.

From 58 high-quality reconstructed MAGs, key players such as *Bifidobacterium subtile IE007* and *Eubacteriaceae IE027*, associated with acetate, butyrate, and ethanol production, were favored at a pH of 6.5. However, a slight pH decreases to 6.1 shifted metabolic activity, promoting caproate and H_2_ production, primarily mediated by *Eubacteriaceae IE037*. The study also demonstrated the feasibility of achieving high ethanol titers (comparable to those from pure yeast cultures) using non-pretreated open-mixed cultures, highlighting the potential for cost-effective bioethanol production from waste. The research further revealed that microbial metabolisms can be modulated to obtain targeted products and that pH alteration can modify the AD process to help the production of valuable market intermediate compounds over biogas. This study underscored the importance of GCM in understanding MC interactions and dynamics, revealing how factor adjustment can shape microbial metabolism to generate added-value products, offering a promising approach to sustainable biorefinery development.

Table 5 presents a detailed comparison of the advanced metagenomics (GCM) tools employed in AD research. It organizes them by the specific processes they support and provides insights into their suitability, innovation, computational demand, and user-friendliness.

**Table 5:** Comparison of GCM tools in AD studies.

Process	Becker et al. (2023)	Greses et al. (2023)	Gaspari et al. (2024)	Overall Suitability	Innovation	Computational Intensity	User-Friendliness
Read Alignment	Bowtie2 v2.3.3.1	Bowtie2 v2.2.4	Bowtie2 v2.4.5	Highly Suitable: Bowtie2 (all versions)	Low: Standard but effective	Medium: Increase with dataset size	High: Well-documented and user-friendly
MAGs Assembly	SPAdes v3.11.1, Canu v1.9, Unicycler v0.4.8	MEGAHIT v1.1.1	MEGAHIT v1.2.9	Highly Suitable: SPAdes, MEGAHIT Suitable: Canu (strong for long-reads)	High: if Canu + MEGAHIT cutting-edge Medium: SPAdes is reliable	High: Canu Medium: MEGAHIT	Medium: SPAdes is user-friendly; Canu requires more expertise
Gene Prediction	Metaxa v2.1.3, Prodigal V2.6.3	Prodigal v2.6.2	Prodigal v2.6.3	Highly Suitable: Prodigal the top choice	Medium: Prodigal, well-established tool	Low: Lightweight	High: Easy to use, widely adopted
MAGs Binning	MaxBin v2.2.7, Concoct v1.1.0, MetaBAT v2	MetaBAT v1.2.15, MetaBAT2 v2.2.15, Concoct v1.1.0, MaxBin2 v2.2.7, VAMB v3.0.2	MetaBAT2 v2.12.1, MaxBin2 v2.2.6, Concoct v1.1.0	Highly Suitable: Multi-tool Suitable: MaxBin alone	High: Multi-tool improves accuracy; Concoct adds binning robustness	High: Multi-tool is computationally intensive; Concoct alone is moderate	Medium: Concoct is user-friendly; MaxBin alone is simpler
MAG Quality Assessment	QUAST v4.5 CheckM	QUAST v3.1, CheckM v1.0.3	CheckM v1.1.3	Highly Suitable: CheckM + QUAST Suitable: CheckM alone	Medium: QUAST is standard; CheckM is well-established	Medium: Moderate resources	High: User-friendly and widely used
MAG Refinement	refineM v0.1.1, MetaWrap v1.2.1, Unicycler v0.4.8	Not reported	Not reported	Highly Suitable: MetaWrap + Unicycler	High: Modern approach to MAG improvement	High: Computationally intensive, multi-step process	Medium: More complex, requires expertise
MAG Dereplication	dRep	dRep v3.2.2	DAS v1.1.2	Highly Suitable: dRep or DAS	Medium: Established tools	Medium: Moderate resources	High: User-friendly
Taxonomic Assignment	GTDB-Tk v1.0.2	GTDB-Tk v1.7.0	GTDB-Tk v2.1.0	Highly Suitable: GTDB-Tk (latest); Suitable: Older versions	Medium: Incremental improvements	Medium: Moderate resources	Medium: User-friendly with good documentation
Phylogenetic Analysis	Not reported	Not reported	PhyloPhlAn v3.0.51, iTOL v6.5.8	Highly Suitable: PhyloPhlAn + iTOL; Suitable: PhyloPhlAn alone	Medium: iTOL adds visualization; PhyloPhlAn is robust	Medium: PhyloPhlAn is moderately intensive, iTOL is light	High: iTOL is user-friendly; PhyloPhlAn requires some expertise
Pathways and Functional Annotation	DIAMOND v0.9.8, MEGAN5 and 6 v6.12.0, Prokka v1.0.2, Prodigal v2.6.3, KEGG, EggNOG, HUMAnN 2.0	METABOLIC, Prodigal v2.6.2, KEGG	Prodigal v2.6.3, EggNOG v2.1.9, HTSeq v2.0.2, KEGG, MicroAnnotator v2.0.4	Highly Suitable: METABOLIC+KEGG; HTSeq, MicroAnnotator Advance Function Annotation; Multi-Tool Suitable: DIAMOND, MEGAN, EggNOG, HUMAnN	High: METABOLIC, HUMAnN2.0 Medium High: MicroAnnotator, HTSeq Medium Low: KEGG, EggNOG	High: METABOLIC, HUMAnN, HTSeq for large datasets Medium: MicroAnnotator	High: Prokka, MEGAN, DIAMOND, Prodigal Medium Low: METABOLIC and HUMAnN need expertise
Community Simulation	Not reported	gapseq v1.1, Micom v0.10.1, Cplex v12.8.0.0	Not reported	Highly Suitable: gapseq + Micom for advanced simulations	High: Cutting-edge for community-level predictions	High: Computationally intensive for complex simulations	Medium: Requires expertise to implement

Recent studies in GCM applied to AD illustrate distinct approaches to the field, showing an evolution towards more refined and comprehensive tools. Becker et al. (2023) used well-established methods, Greses et al. (2023) joined traditional and cutting-edge techniques, while Gaspari et al. (2024) pushed the boundaries with innovative approaches. This progression reflects a pattern of evolution in metagenomics, with a trend toward increasingly sophisticated and computationally intensive tools that offer higher accuracy and detail.

Unlike 16S rRNA metataxonomics, generally limited to genus-level taxonomic resolution and lacking in functional detail, being confined to known organisms within reference databases, and often unable to detect novel microbes, these findings demonstrate the importance of furthering metagenomics through GCM. Using long, high-quality reads, GCM has confirmed the association of specific organisms with essential catabolic pathways, optimizing these routes under suboptimal conditions. For example, syntrophic interactions involving *Syntrophobacter* and *Syntrophomonas* are integral to the degradation of short-chain fatty acids and biogas production. Moreover, hydrogenotrophic methanogenesis becomes more prominent under ammonia stress, with *Methanoculleus* species contributing to system resilience.

GCM’s ability to uncover novel genomes missed by short-read studies and reveal new functional competencies refines the understanding of microbial metabolic diversity, challenging the conventional four-step AD scheme. As GCM techniques advance, they provide more accurate predictions of biogas production, transforming AD from a “black box” into a more manageable system. However, the increased accuracy and detail come at a higher cost, requiring a balance between precision and resources in sustainable biorefinery development. This overview helps researchers align established methods with cutting-edge techniques, highlighting the growing sophistication of metagenomics in unraveling the AD microbiome.

### 5.2 Strain-resolved metagenomics applied to biogas production

Although the recovery of high-quality MAGs denotes a substantial advancement in GCM within AD research, underexplored layers of microbial genome diversity remain. These hidden layers require more sophisticated methodologies to fully elucidate comprehensive microbial adaptive strategies, surpassing the limitations of species-level resolution (Greses et al., 2023; Ghiotto et al., 2024a). For instance, advanced microbiome evolutionary, functional, and metabolic tracking mechanisms are essential. Such a mechanism would enable the selection of microorganisms at a higher level of diversity through metagenome strain identification (Bucci et al., 2024; Ghiotto et al., 2024a), recognizing strains as the fundamental unit of microbiological diversity, a group of single-cell clonal descendants (Quince et al., 2021; Setubal, 2021). This approach allows for monitoring diversity beyond the species level, with the capacity to detect genetic changes even without microbial abundance fluctuations (Ghiotto et al., 2024b).

In AD research, merely evaluating species dynamics often falls short of capturing the nuanced changes within species and the temporal succession of coexisting strains (Bucci et al., 2024; Ghiotto et al., 2024b). Single-nucleotide variants (SNVs) analysis emerges as a promising approach to bridge this gap. This method permits the detection of SNVs at specific genomic positions, whether within protein-coding sequences or intergenic regions (Zou et al., 2020). By revealing shared gene variations, such as insertions and deletions, SNV analysis makes it possible to track strains within a species based on allele pattern detection (Quince et al., 2021; Bucci et al., 2024; Ghiotto et al., 2024b).

A complementary strategy known as strain deconvolution uses shotgun metagenomics reads to concurrently determine strain genotypes and relative abundances across samples. This is accomplished via statistical deconvolution of allele frequencies, a process that separates mixed genetic signals into their parts, allowing the identification of multiple genotypes from pooled data (Smith et al., 2022). This procedure has proven effective in evaluating the genetic dynamics and ecological fluctuations of AD species, especially in tracking emerging mutations at species and strain levels (Bucci et al., 2024; Ghiotto et al., 2024b). These interrelated methods allow the tracking of microbial strains, assessment of their relative abundances, identification of dominant organism variations, and monitoring of evolutionary trends, thereby aiding in the identification of suitable environmental conditions for archaea and bacteria syntrophies during AD (Ghiotto et al., 2024b).

Bucci et al. (2024) explored the impact of escalating ammonia concentrations on MC within AD. The study sought to delve deeper into microbial evolution by applying SRM, focusing on monitoring SNVs over time. By gradually increasing ammonia levels across cultivation generations, the study aimed to unravel the dynamics of ammonia-tolerant methanogenic consortia at both species and strain levels. Strain deconvolution was employed to isolate genetic variants from shotgun metagenomics data, providing a granular view of allele occurrences. This approach enabled a detailed examination of how microbial consortia adapt to increasing ammonia stress at a finer genetic resolution.

The investigation identified 179 MAGs (172 bacterial and 7 archaeal), with *Firmicutes* as the predominant bacterial phylum. Among the archaeal species, *Methanoculleus bourgensis vb3066* was notably abundant, likely due to its syntrophic interactions with bacteria involved in the Wood-Ljungdahl (WL) pathway, a carbon fixation process crucial for converting CO_2_ to acetate, and the glycine synthase reductase pathway. SAOBs like *Keratinibaculum* sp. *ma44* and *Acetomicrobium* sp. *ma133* were also key players. Over time, the relative abundance of *Clostridium cochlearium ma73*, *Keratinibaculum* sp. *ma44*, and *M. bourgensis vb3066* increased, while *Acetomicrobium* sp. *ma133* and *Firmicutes* sp. *ma48* showed reduced relative abundance at later generations, possibly because of competition and ammonia tolerance thresholds. The study identified *Acetomicrobium* sp. *ma133, C. cochlearium ma73, Firmicutes* sp. *ma48, Firmicutes* sp. *mb175, Keratinibaculum* sp. *ma44, Keratinibaculum* sp. *mb43,* and *M. bourgensis vb3066* as key organisms capable of thriving in high ammonia conditions. These findings accentuate the importance of specific microbes in maintaining metabolic balance under challenging conditions.

Notably, 148 non-synonymous SNVs were identified in enzymes associated with the WL and glycine synthase reductase pathways in *Acetomicrobium* sp. *ma133148*, indicating significant selective pressures that allowed these enzymes to maintain their functionality under elevated ammonia concentrations. The persistence and dominance of *M. bourgensis vb3066* and *Acetomicrobium* sp. *ma133* suggest that these strains have adapted to stabilize the system by mitigating inhibition and fostering robust syntrophic relationships. Genes associated with the WL and glycine synthase reductase pathways in *C. cochlearium ma73*, *Keratinibaculum* sp. *ma44*, and *Tepidanaerobacteraceae* sp. *ma135* might imply that all or some of these organisms may have taken on roles as putative SAOB (Bucci et al., 2024). Strain deconvolution was thus proven to be an effective technique for identifying intraspecific diversity, displaying coexisting strains with diverse phenotypes as organisms respond uniquely to environmental pressures. This genetic evidence emphasizes the critical role of SNVs in supporting microbial adaptation and survival, reinforcing the importance of genetic diversity.

Recognizing the need to improve AD products, such as increasing CH_4_ content and reducing H_2_S concentration, Ghiotto et al. (2024a) studied the evolution of a mixed-methanogenic culture using a novel approach involving variant calling and strain deconvolution. The research subjected the methanogenic community to high H_2_S levels in a trickle bed reactor, a type of packed bed reactor that facilitates interaction between solid, liquid, and gas phases during chemical reactions driven by gravity-assisted flow (Malolan et al., 2022). The experiment was divided into three stages: S1 (artificial and sulfur-rich biogas), S2 (pure H_2_ with biogas from a lab-scale continuous stirred-tank reactor), and S3 (microaerophilic conditions with 0.002% v/v O_2_). These stages aimed to evaluate the combined biomethanation and desulfurization processes of anoxic and limited oxygen conditions while assessing the metabolic capabilities of AD microbiota to achieve biogas upgrading as a sustainable alternative to conventional chemical methods.

Throughout the study, 97 to 146 high-quality MAGs were identified, revealing 171,545 distinctive SNVs across six samples, 37% of which were nonsynonymous. In S1, the microbial landscape was prominently shaped by *Methanobacterium* sp. *DTU45, a* hydrogenotrophic methanogen that prevailed under sulfur-enriched conditions due to its capacity to utilize the elevated H_2_ levels. Its biofilm-forming ability could have offered protection against toxic agents like oxygen, further contributing to its resilience. Concurrently, *Gammaproteobacteria* sp. *DTU53* began to proliferate, capitalizing on the controlled conditions. This initial phase provided critical insights into the early adaptive mechanisms of the methanogenic community when exposed to elevated H_2_S levels. During S2, putative SOB such as *Gammaproteobacteria* sp. *DTU53* benefited from a gas injection, with an alteration in abundance indicating a possible electron redirection to the sulfate-sulfur receptor pathway. In S3, 81% of H_2_S was successfully removed, and *Gammaproteobacteria* sp. *DTU53* reappeared, potentially exhibiting a metabolic modification from fermentation to aerobic respiration, utilizing sulfur compounds as electron donors. Facultative anaerobes like *A. equifetale DTU58* also contributed to desulfurization as a potential oxygen-resistant species in this microaerophilic environment. Additionally, the identification of a molecular marker for potential SAOB, the formate-tetrahydrofolate ligase gene, in *Limnochordia* sp. *DTU66*, *Proteinivoracia* sp. *DTU68*, and *Firmicutes* sp. *DTU69*, participants in the WL pathway, could have encouraged facultative syntrophies with *Methanobacterium* species.

The study demonstrated that genomic variant growth persuades phenotypic changes, improving functional redundancy and resilience within the microbiome. Strain deconvolution effectively revealed intraspecific diversity, allowing for successful microaerophilic biomethanation (95% CH_4_) and hydrogen sulfide removal (81%), without hindering hydrogenotrophic archaea. However, while these findings are promising, the controlled experimental conditions may not fully reflect the complexities of industrial-scale systems. The scalability and wider applicability of these results required additional validation in more varied and realistic environments.

Seeking to promote sustainable practices within the Carbon Capture and Utilization concept, Ghiotto et al. (2024b) focused on achieving biological biogas upgrading through autotrophic approaches. The study highlighted the relevant role of hydrogenotrophic archaea in balancing H_2_ levels while cooperating with sulfate-reducers and SAOB to remove acetate and sulfur. Given the limited knowledge of SNVs’ impact on hydrogenotrophic archaea, this research analyzed how SNVs influence the functional properties of these organisms for biomethane production, aiming to uncover new genetic insights.

Two case studies were conducted: the first assessed various feedstock substrates, while the second evaluated the effect of continuous H_2_ addition on the microbiota. In the first experiment, 47 high-quality MAGs were identified, with *Firmicutes* dominating at 93% abundance. *Methanothermobacter wolfeii MA_1* was notably abundant, primarily using the CO_2_ to CH_4_ reduction pathway and acetate, formate, and methanol when H_2_ was unavailable. *Sphaerobacter thermophilus CO_9*, *Caldanaerobacter subterraneus MX_27,* and *Limnochordia* sp. *MA_37* were the predominant bacteria.

The second experiment yielded 50 high-quality MAGs, with *Firmicutes* as the most abundant bacteria. *Methanosarcina thermophila MB_65* and *Methanocullus thermophilus MA_62* were the dominant archaea, each with distinct metabolic preferences: acetate for *M. thermophila* and H_2_ for *M. thermophilus*, which thrived with H_2_ addition. *Limnochordia* sp. *MB_100*, *Bacteroidales* sp. *VB_122*, and *Acetomicrobium* sp. *MX_67* showed the highest relative abundance.

It is likely that syntrophic relationships between acetate-oxidizing bacteria, *M. wolfeii MA_1*, and *M. thermophilus MA_62* might have promoted the high abundance of both archaea. A total of 76,229 SNVs were identified and categorized as synonymous (56%), nonsynonymous (30%), and intergenic (14%) variations. Analysis of relative abundance and SNV accumulation over time indicated that species dominance was linked to a higher number of genetic variants. Defined environmental parameters facilitated cooperative coexistence within the MC during methanogenesis, involving conventional and alternative WL pathways. Notably, it is suggested that the efficiency of carbon capture and utilization within MC be prioritized over isolation experiments to enhance biogas production by identifying the most efficient and resilient strains (Ghiotto et al., 2024b).

Table 6 provides a clear and concise comparison of the field’s current state in strain-level analysis in AD research, showcasing key tools and their applications. This overview, grounded in insights from rigorous and recent studies, serves as a valuable guide for researchers in selecting suitable techniques for their specific research needs.

**Table 6:** Comparison of the current tools used for strain-level analysis in AD research.

Process	Bucci et al. (2024)	Ghiotto et al. (2024a)	Ghiotto et al. (2024b)	Overall Suitability	Innovation	Computational Intensity	User-Friendliness
SNV Identification and Analysis	InStrain v1.6.3	InStrain v1.6.3	InStrain v1.6.3	Highly Suitable: Specialized for SNV analysis	High: Focused on SNV identification	Medium: Moderate computational demand	Medium: Requires some expertise
Variant Clustering	Mann–Whitney U Test	No reported	No reported	Highly Suitable: Accurate variant clustering	Medium: Traditional statistical approach	Low: Low computational demand	Medium: Moderate difficulty in implementation
Strain Resolution and Deconvolution Pipeline	STRONG	STRONG	STRONG	Highly Suitable: Accurate strain resolution	High: Novel approach with BayesPaths algorithm	High: Computationally intensive due to graph disentangling	Low: Complex, requires advanced expertise
3D Protein Structure Modeling	No reported	AlphaFold	No reported	Highly Suitable: Cutting-edge for protein modeling	High: Advanced AI-based modeling	High: Computationally intensive	Medium High: Requires expertise to use effectively
Protein Sequence Alignment	No reported	Clustal Omega	No reported	Suitable: Standard for sequence alignment	Low: Established	Medium: Moderate computational demand	High: User-friendly, well-documented
Graph Disentangling	No reported	No reported	BayesPaths	Highly Suitable: Specialized for complex graph disentangling	High: Innovative and complex	High: Computationally intensive	Low: Requires advanced expertise
SNV Clustering Analysis	No reported	No reported	Python (scipy.stats)	Suitable: Versatile for statistical analysis	Medium: Flexible and adaptable	Medium: Depends on dataset size	Medium: Requires programming knowledge
Gene annotation and Metabolic reconstruction	Prodigal v2.6.3, eggNOG-mapper v2.1.9, KEGG, Microbe-Annotator v2.0.4	Prodigal v2.6.3, eggNOG-mapper v2.1.9, KEGG, Microbe-Annotator v2.0.4	Prodigal v2.6.3, eggNOG-mapper v2.1.9, KEGG	Highly Suitable: Multi-tool comprehensive annotation and reconstruction	Medium High: Microbe-Annotator Medium: Established and reliable tools	Medium: Moderate computational demand	Medium: Requires some expertise
Phylogenetic analysis	Phylophan v3.0, iTOL	No reported	No reported	Highly Suitable: Robust for phylogenetic analysis	Medium: Establish method	Medium: Moderate computational demand	Medium: Requires some expertise

InStrain v1.6.3 is a widely used tool for SNV identification, known for its precision, though it requires moderate computational resources and expertise (Lindner et al., 2024). STRONG, integral to strain resolution, delivers high accuracy via its BayesPaths algorithm but is computationally intensive and complex to implement (Quince et al., 2021).

AlphaFold, used in one study for 3D protein modeling, provides cutting-edge insights but is also demanding in terms of computational power. Gene annotation relies on well-established tools, with Microbe-Annotator v2.0.4 being the most innovative, providing comprehensive analyses but with considerable computational requirements. For phylogenetic analysis, PhyloPhlAn v3.0 and iTOL are reliable options, requiring moderate computational power. The choice of tools varies in complexity, computational load, and user-friendliness, often demanding substantial expertise to achieve optimal results.

The exploration of advanced GCM and SRM in AD research represents a shift from species-level analysis to more refined strain-level insights. Studies by Ghiotto et al. (2024a), Bucci et al. (2024), and Ghiotto et al. (2024b) used SRM to reveal hidden layers of microbial genome diversity that traditional 16S rRNA gene-based metataxonomics or even GCM could not fully capture. SRM’s ability to track SNVs and analyze their influence on functional and metabolic traits provides a more comprehensive understanding of microbial adaptation to stressors such as high ammonia or H_2_S levels.

These studies consistently identified resilient strains of hydrogenotrophic archaea, which maintained metabolic balance through DIET and biofilm formation. A common theme was the discovery of numerous SNVs contributing to phenotypic variation, species dominance, increased functional redundancy, and overall microbial success under various conditions.

Implementing SRM and high-quality MAG recovery involves substantial computational and sequencing costs compared to 16S rRNA gene analysis. The need for deep sequencing, extensive computational resources for SNV analysis, and specialized software for strain deconvolution all contribute to higher expenses. Nevertheless, the added cost is justified by the richer, more actionable insights that SRM provides for optimizing AD operations.

Variant analysis grants critical perceptions into AD metabolic pathways by revealing how microbial evolution and strain dominance occur. Nonsynonymous SNVs can influence microbial metabolism and functional potential by altering protein structures, leading to gains or losses in function that can enhance biogas yields. The strain-level resolution provided by SRM is essential for knowing the impacts of selective pressures on MC structure and elucidating the adaptive mechanisms that support stable bioconversion and resistance development in inhibitory environments.

While GCM is valuable for understanding community compositions, it often lacks the precession needed to capture strain-level diversity and the functional implications of SNVs. In contrast, SRM offers novel insights into the evolution and interactions of AD microbiota, particularly under adverse conditions. Future efforts should prioritize identifying and enhancing the most robust and efficient strains within diverse MCs, ensuring that detailed genetic knowledge is applied with cost-effectiveness and wider practical implementation.

## 6 Challenges and perspectives of metagenomics in AD research

In AD research, replication is frequently neglected due to cost and time constraints, yet it is essential for ensuring statistical robustness and improving pattern detection in microbial ecology. This lack of replication is especially problematic in metagenomic studies, where sampling and storage biases can distort MC analysis. Additionally, the incomplete nature of metagenomics data hinders the identification of functional genes and the detection of uncharacterized microorganisms in biogas samples. Expanding gene databases is crucial to address these limitations, providing stronger evidence for functional predictions and capturing microbial diversity more effectively.

Combining GCM with SRM could offer a robust approach to refining functional predictions and exploring ecological interactions within complex MC (Kim et al., 2022). GCM reconstructs MAGs, providing insights into the genetic makeup and functional potential of different microbial populations in a community. However, it may not distinguish between closely related strains within a species. SRM addresses this limitation by resolving the strain level and uncovering the genetic diversity and specialized functions that dive into processes like organic matter degradation and methane production. Together, they might display a profound perspective on community-wide functions and the distinct contributions of individual strains.

Further integration of advanced metagenomics with multi-omics and molecular technologies would provide new depth in understanding MC functions (Kim et al., 2022; Li et al., 2022). This integration enhances the accuracy of functional predictions and allows for more precise manipulation of microbial consortia to achieve desired outcomes in AD processes, such as efficiently producing biochemicals and biofuels, supporting the shift toward sustainable production systems (Greses et al., 2023).

Molecular methods permit a detailed exploration of microbial ecological roles and metabolic capabilities through synergistic networks supported by catabolic complementarity, electron transfer balance, and energy conservation. In this context, the promising scientific discipline, ecogenomics, which studies the relationships between microbial functions and their environments, is valuable (Nobu et al., 2015; James and Richardson, 2020).

Concepts such as quorum sensing, a microbial cell-to-cell signal molecules communication system bridging biological processes between MC by regulating gene expression, population behavior, and density via extracellular autoinducers (Zhang et al., 2021; Wang et al., 2023), should be considered in microbial connectivity techniques in AD. This system can regulate AD metabolic processes such as chemical oxygen demand removal and methanogenesis (Lv et al., 2023). Similarly, metagenomics could become a useful tool for identifying microbiota with an affinity for conductive materials, thereby promoting DIET and enhancing CH_4_ production (Lei et al., 2019). A deeper understanding of microbiome interactions will lead to more efficient methodologies to sustain key metabolic routes in AD.

Metagenomic analysis through large-scale sequencing faces significant challenges, including substantial computing requirements, limited storage, extended processing times, and high sequencing costs. To address these, integrating supercomputing with data mining and ML algorithms on cloud platforms is essential. These algorithms should be specifically designed for metagenomics and linked to open-source databases to improve accuracy (Zhang et al., 2019a; Yang et al., 2021). However, AI methods in metagenomics are still constrained by high computational demands and the need for large, well-annotated datasets, which are often lacking in complex environments like AD. This shortage can lead to bias and generalization challenges. Therefore, enhancing data curation and optimizing models are essential for the wider adoption of advanced AI-driven approaches.

The future of AD research may involve developing specialized tools within more user-friendly platforms, combining precision with ease of use. This would make technologies accessible to a wider range of researchers and practitioners. Additionally, using real-time metagenomic data alongside operational controls could allow for dynamic adjustment to process parameters, enhancing system resilience and efficiency. This approach provides a comprehensive view of the functional potential within microbial populations, enabling more responsive and efficient management.

In summary, metagenomics has significantly advanced the analysis of microbial communities (MC) in anaerobic digestion (AD), but further progress in technology and methodology—particularly in real-time monitoring, multi-omics integration, and machine learning—will be crucial for optimizing these systems. Addressing these current challenges in sampling, data handling, computational demands, and data completeness while continuously expanding gene databases and providing direct evidence for predicted functionalities, will be key to fully unlocking the potential of metagenomics in AD research.

## 7 Conclusion

This review has thoroughly explored the transformative potential of metagenomics in revolutionizing AD processes. By providing a detailed understanding of the microbiome involved, metagenomics has clarified the crucial role of MCs in converting residual biomass into valuable products such as biogas, biomethane, biochemicals, and biofuels. The review also highlights how feedstock composition and operational conditions significantly shape MC structure and function.

Metagenomics is a powerful tool for identifying and characterizing entire microbiomes, including non-cultivable organisms while providing valuable insights into their metabolic pathways and interactions. The integration of advanced techniques, such as GCM and SRM, has further deepened the understanding of microbial dynamics, evolution, adaptations, and functional capabilities within AD systems.

This enhanced understanding of MC interactions paves the way for targeted interventions and process optimization, helping to unravel the complexity of the AD system. Identifying and isolating key MCs, combined with the development of user-friendly tools and real-time monitoring systems, holds immense promise for optimizing AD operations, achieving higher biogas yields, and ultimately developing more efficient and sustainable AD technologies. By empowering researchers and practitioners with critical insights, the application of metagenomics is poised to play a pivotal role in advancing the AD field.
